# Native Polyethylene Biodegradation by Gut-Associated Bacteria from *Galleria mellonella* Larvae: Multi-Method Evidence Without Abiotic Pretreatment

**DOI:** 10.3390/polym18182309

**Published:** 2026-09-21

**Authors:** Óscar Martínez González, Mirelys Cáceres, Ángel Grajales, Luis González, Franz Robles, Mariel Monrroy, Nicomedes Ariel Jaramillo, Aurelio Boya, Roberto Guevara, Jorge Ceballos, Alex Ríos-Moreno

**Affiliations:** 1 Department of Biology, Faculty of Natural and Exact Sciences, Autonomous University of Chiriqui, David 04001, Panama; mirelys.caceres@unachi.ac.pa (M.C.); angel.grajales@unachi.ac.pa (Á.G.); luis.gonzalez2@unachi.ac.pa (L.G.); franz.robles@unachi.ac.pa (F.R.); 2 Agri-Food Biotechnology and Applied Physics Research Group (BIOFISAGRO), David 04001, Panama; aurelio.boya@unachi.ac.pa; 3 Natural Products and Biotechnology Research Center (CIPNABIOT), Faculty of Natural and Exact Sciences, Universidad Autónoma de Chiriquí (UNACHI), David 04001, Panama; nicomedes.jaramillo@unachi.ac.pa (N.A.J.); roberto.guevara@unachi.ac.pa (R.G.); 4 Department of Chemistry, Faculty of Natural and Exact Sciences, Autonomous University of Chiriqui, David 04001, Panama; mariel.monrroy@unachi.ac.pa; 5 National Research System (SNI), National Secretariat of Science, Technology and Innovation (SENACYT), Panama City 0816-02852, Panama; 6 Research Center in Biochemistry and Applied Chemistry, Faculty of Natural and Exact Sciences, Autonomous University of Chiriqui, David 04001, Panama; 7 Department of Physics, Faculty of Natural and Exact Sciences, Autonomous University of Chiriqui, David 04001, Panama; 8 Laboratory of Biosciences, Biotechnology, and Applied Sciences, Faculty of Science and Technology, Technological University of Panama, David 04001, Panama; jorge.ceballos1@utp.ac.pa; 9 Plant Health Department, Faculty of Agricultural Sciences, University of Panama, David 04001, Panama

**Keywords:** *Acinetobacter pittii*, AlkB, *Enterobacter hormaechei*, entomoremediation, *Galleria mellonella*, gut microbiome, Polyethylene biodegradation

## Abstract

The accumulation of polyethylene (PE) in the environment represents a persistent ecological challenge, and the search for biological agents capable of degrading it without relying on prior abiotic pretreatment remains an active area of research. In this study, three bacterial strains—*Pseudomonas aeruginosa*, *Enterobacter hormaechei*, and *Acinetobacter pittii*—were isolated from the gut of *Galleria mellonella* larvae dietarily induced with native low-density polyethylene (LDPE), without prior UV, thermal, or chemical treatment. Molecular identification was confirmed by 16S rRNA gene sequencing and maximum-likelihood phylogenetic analysis. Degradative activity was assessed using a multi-method approach: a colorimetric DCPIP assay, gravimetric mass loss at 45 days, FTIR-ATR spectroscopy, SDS-PAGE, and scanning electron microscopy. All three strains showed metabolic activity toward LDPE, with *A. pittii* producing the greatest mass loss. FTIR analysis revealed the appearance of hydroxyl and C–O-containing functionalities without detectable carbonyl accumulation, a pattern consistent with the hypothesized activation of an oxidative pathway mediated by the alkane hydroxylase (AlkB) system, pending direct molecular/biochemical confirmation. These results expand the still-limited number of reports on native-PE-degrading activity documented individually for *E. hormaechei* and *A. pittii* and reinforce the value of the *G. mellonella* gut microbiome as a reservoir of bacteria with biotechnological potential for plastic waste management.

## 1. Introduction

Plastic pollution represents one of the most pressing environmental challenges of the 21st century. It is estimated that more than 400 million tons of plastic are produced each year globally, a significant fraction of which ends up fragmenting into microplastics (particles < 5 mm) and nanoplastics (<1 µm) that persist in terrestrial, aquatic, and marine ecosystems for hundreds of years [1,2]. Polyethylene (PE) is the most widely produced synthetic polymer worldwide and one of the main components of household and industrial plastic waste, characterized by its high resistance to chemical and biological degradation, which is attributed to its covalent carbon–carbon σ bonds.

The ubiquity of microplastics has transcended natural ecosystems to directly reach the human food chain. Recent studies have documented the release of micro- and nanoplastics from nylon, polypropylene, and PET during standard preparation of commercial tea bags, with confirmed internalization in human intestinal cells [3]. This finding, together with the detection of microplastics in drinking water, table salt, fish, and human tissues, underscores that exposure to microplastics is not solely an environmental phenomenon but a public health issue with toxicological implications that are not yet fully understood. The urgency of developing effective, sustainable, and field-applicable degradation strategies has driven growing interest in the microbial biodegradation of synthetic polymers as an alternative to conventional physical and chemical methods.

Microbial biodegradation of PE is a slow and complex process involving surface colonization of the polymer, secretion of extracellular oxidative enzymes, and progressive assimilation of the generated fragments. Various bacterial species have been reported as PE degraders, including *Pseudomonas aeruginosa* [4,5], *Bacillus subtilis*, and members of the genera Rhodococcus and Streptomyces [2]. However, the vast majority of these studies use PE pretreated by UV irradiation, heat, or oxidative chemical treatments as a prerequisite for bacterial exposure, which significantly limits the extrapolation of their results to real environmental conditions. Degradation of native PE by bacteria without prior abiotic pretreatment has been documented only to a very limited extent, constituting one of the main gaps in the field [2,6].

In this context, xylophagous and plasticophagous insects have emerged as high-value biological models for the bioprospecting of polymer-degrading microorganisms. The gut of these insects functions similarly to a natural selection bioreactor: conditions of partial anaerobiosis, acidic pH, constant temperature, and, crucially, the selective pressure imposed by a diet rich in synthetic polymers generate a microenvironment that actively enriches for bacteria with catabolic capacity toward hydrophobic substrates with high activation energy. Unlike direct isolation from contaminated soils, gut transit in the insect acts as an in vivo enrichment process that concentrates and functionally selects bacterial taxa with the greatest activity on the polymer. This principle has been demonstrated in *Tenebrio molitor* [1] and in the wax moth larva *Galleria mellonella* [7], forming the conceptual basis of the approach known as entomoremediation.

*Galleria mellonella* (Lepidoptera: Pyralidae), known as the wax moth, exhibits an exceptional metabolic predisposition for polyethylene degradation, derived from its natural ability to catabolize beeswax, a substrate chemically analogous to PE in terms of its aliphatic hydrocarbon chains. A previous study by our group [8] documented the in vivo oxidation of low-density polyethylene (LDPE), expanded polystyrene, and oxo-biodegradable plastics by *G. mellonella* larvae, evidenced by an increase in the carbonyl index of the polymers recovered from the digestive tract. This oxidation was attributed predominantly to the host’s own enzymes—intestinal proteinases and oxidases—raising the unresolved hypothesis of whether the bacteria of the gut microbiome additionally possess their own enzymatic machinery with independent degradative capacity.

The present study arises directly from this open question. The gut microbiome of *G. mellonella* may represent more than a passive companion in the digestive process: a bacterial community shaped by the evolutionary pressure of the substrate, capable of synergistically enhancing polyethylene degradation or of exerting it entirely on its own. Therefore, the objective of this work is to determine whether bacteria isolated from the gut of *G. mellonella* larvae exhibit biodegradative activity toward low-density polyethylene (LDPE).

## 2. Materials and Methods

*G. mellonella* larvae were obtained in the Agri-Food Biotechnology and Applied Physics laboratory at UNACHI (BIOFISAGRO) and distributed into four experimental groups according to diet (Table 1). Induction with low-density polyethylene (LDPE) powder (500 mesh; Keyue Material Store, Dongguan, China) was maintained for three months. Feed was replaced every 25–30 days to prevent fungal growth associated with the humidity generated by larval activity and to ensure constant nutritional conditions throughout the experiment.

### 2.1. Isolation and Recovery of Bacterial Strains

At the end of the induction period, ten randomly selected L6-instar larvae (*n* = 10) from each treatment were used. Guts were aseptically excised, macerated, and resuspended in 5 mL of sterile 0.9% saline solution. The suspension was centrifuged at 4000 rpm (~1789× *g*) for 5 min (TGL-16 centrifuge, Hunan Xiangyi Laboratory Instrument Development Co., Ltd., Changsha, China), and 1 mL of the supernatant was inoculated into enrichment media: brain–heart infusion (BHI) broth, tryptic soy broth (TSB), and thioglycolate broth, incubated at 35 °C for 24 h (DH3600IIB series incubator, Faithful Instrument (Hebei) Co., Ltd., Huanghua, China). Subsequently, cultures were plated onto selective media: MacConkey agar, blood agar, and Cetrimide agar, and incubated at 35 °C for 24–48 h. Four colonial morphotypes with distinguishable macroscopic characteristics were selected. Selected colonies from the treatment and control groups were inoculated into LB broth and incubated at 35 °C for 24 h for subsequent characterization. In total, five different isolates were obtained: UNKNOWN0, UNKNOWN1, UNKNOWN8, UNKNOWN9, and UNKNOWN10.

Morphotype selection was based on macroscopic diversity after isolation on general and selective culture media, without prior in vitro screening based on the use of LDPE as the sole carbon source, given that the particulate and opaque nature of the polymer does not allow visual detection of degradation halos on plates. The biodegradative capacity of each isolate was subsequently evaluated, once identified, using the DCPIP assay (Section 2.5) and the gravimetric assays (Section 2.6). This approach rests on the premise that the dietary selective pressure sustained over three months in the larvae—rather than an additional in vitro screening step—constituted the main enrichment mechanism for bacteria with catabolic activity toward the polymer.

### 2.2. Bacterial Identification by Biochemical Tests

Preliminary identification of the isolates was carried out using the following biochemical tests: triple sugar iron (TSI), indole production, motility and H_2_S production (SIM), Voges–Proskauer (VP), urea utilization, and Simmons citrate, all incubated at 35 °C for 24 h, as described by MacFaddin and Koneman et al. [9,10].

### 2.3. Molecular Identification by 16S rRNA Gene Sequencing

Molecular identification was carried out by 16S rRNA gene sequencing. Total DNA was extracted, and the 16S rRNA gene was amplified by PCR using the universal primers 27F (5′-AGAGTTTGATCMTGGCTCAG-3′) and 1492R (5′-TACGGYTACCTTGTTACGACTT-3′), generating an amplicon of approximately 1500 bp. Sanger sequencing was performed by Macrogen Inc. (Seoul, Republic of Korea), orders HC01300059 and HC01316577. The sequences obtained were compared against the NCBI GenBank database using the BLASTn algorithm versión 2.13.0+. Sequences were deposited in GenBank prior to publication of the article.

### 2.4. Assessment of Metabolic Activity Using the DCPIP Redox Indicator

Metabolic activity toward PE was assessed using the colorimetric 2,6-dichlorophenolindophenol (DCPIP) assay [11], whose color change (from blue in its oxidized form to colorless in its reduced form) reflects the oxidative activity of microorganisms toward the polymer. During degradation, electrons released by oxidation of PE fragments reduce DCPIP, producing a decrease in absorbance at 600 nm that indicates the presence of PEase-type enzymatic activity. Each experimental unit consisted of 0.006 g of DCPIP and 2 g of PE in carbon-free liquid basal medium (CFLBM). The composition of the CFLBM is presented in Table 2. Absorbance was measured at 600 nm on days 1, 4, 5, 6, and 7 of incubation, generating a temporal consumption kinetic. The sampling scheme concentrated on day 1 (baseline) and the interval from days 4 to 7, the window in which the greatest variation in absorbance signal was anticipated, while days 2 and 3 were not sampled; this decision was based on the expectation that the change in absorbance levels during the initial phase (days 2–3) would be minimal, prioritizing sampling resolution during the period of greatest expected change. Two abiotic controls were included: Control 1 (DCPIP + PE, no inoculum) to verify the chemical stability of the indicator, and Control 2 (DCPIP + inoculum, no PE) to quantify baseline metabolic activity. The relative consumption percentage was calculated as percentage (%) consumption = (1 − A_t_/A_0_) × 100 (1), where *A*_0_ is the initial absorbance (day 1) and *A_t_* is the absorbance at time _t_. For first-order kinetic analysis, ln (A_t_/A_0_) was calculated as a function of time, and linear regression was applied to obtain the apparent rate constant (k, day^−1^) and the half-reduction time (t_½_). An interaction model (treatment × day) was applied via multiple linear regression to evaluate whether DCPIP reduction kinetics differed significantly between strains and controls over time. This main DCPIP assay was performed as a single screening run per strain, with no biological replication, given its purpose as a high-throughput tool for prioritizing isolates for downstream characterization; the results should accordingly be interpreted as an indirect indicator of redox/metabolic activity toward PE rather than as a direct, quantitative measure of the extent of polymer degradation.

### 2.5. Bacterial Metabolic Resistance Test

To evaluate the resistance of the putative enzymes involved in PE degradation to abiotic (thermal and oxidative) stress, three treatments were designed in duplicate for each strain. Each strain was subjected to the following pretreatments prior to activity assessment on powdered polyethylene: (A) normal condition (standard culture), (B) exposure to UVC + O_3_ radiation for 15 min using Black Magic UV-C lamps (short-wave ultraviolet light) (G6 Wellness) with a wavelength of 254 nm and power of 25 watts (Santacary Technology Co., Ltd., Shekou, Shenzhen, Guandong, China) and ozone (O_3_)-generating lamps with a concentration of 500 mg/h ≈ 8.33 mg/min, and (C) boiling at 100 °C on a hot plate for 15 min. Once the pretreatments were completed, the assay was carried out using the methodology described in Section 2.5. DCPIP absorbance was measured on days 1, 3, and 5. For visual trend illustration in graphical representations, values for days 6 and 7 under boiling and UV treatments were projected using linear trend extrapolation.

To analyze the combined effects of physical treatments and incubation time on experimental days 1, 3, and 5, a two-way repeated measures ANOVA (treatment × time) was performed individually for each bacterial species using a significance level of α = 0.05. Post hoc pairwise comparisons were carried out using Fisher’s LSD test to identify significant differences between treatments at specific time points (Day 3 and Day 5).

### 2.6. Gravimetric Assessment of Polyethylene Biodegradation

Commercial films of conventional PE and oxo-degradable PE (OXO), without prior pretreatment, in 35 mm diameter discs, were incubated with 2 mL of CFLBM inoculated with each bacterial strain for 45 days at 35 °C (DH360011 model incubator). The inoculum was replaced weekly. At the end of the incubation period, the films were recovered, washed with sterile distilled water, dried, and weighed three times on an analytical balance (HR-250A analytical balance, A&D Company, Ltd., Toshima-ku, Tokyo 170-0013, Japan). Percentage mass loss and daily degradation rate (mg/day) were calculated by weight difference. Statistical significance was assessed using Student’s *t*-test (α = 0.05). The abiotic (sterile) control was incubated under conditions identical to the inoculated treatments: same medium (CFLBM), same temperature (35 °C), static incubation (no agitation), and the same total duration of 45 days, with weekly medium renewal. The only difference between the control and the experimental treatments was the absence of bacterial inoculum, which allows the observed mass loss to be attributed specifically to microbial activity.

(1) $$\mathrm{Degree}\;\mathrm{of}\;\mathrm{Consumption}\;(\%)=\left(\frac{W_{i}\left(g\right)-W_{f}\left(g\right)}{W_{i}\left(g\right)}\right)\times 100\%$$

where

Degree of Consumption:

*W_i_* (*g*): Initial weight in grams;

*W_f_* (*g*): Final weight in grams.

### 2.7. Structural Characterization by FTIR-ATR

Structural changes in the PE and OXO films resulting from Section 2.7 were assessed by attenuated total reflectance Fourier-transform infrared spectroscopy (FTIR-ATR) IRTracer-100 spectrophotometer (Shimadzu, Columbia, MD, USA). Analyses were performed at the Center for Research in Biochemistry and Applied Chemistry (CIBQUIA) of the Autonomous University of Chiriqui, using an IRTracer-100 spectrophotometer (Shimadzu, Columbia, MD, USA) in ATR mode with a Ge crystal, at a resolution of 2 cm^−1^ and 32 scans per spectrum, over the spectral range 4000–600 cm^−1^. Two spectra were acquired per sample and averaged. Samples were washed, dried, and stored in a desiccator prior to analysis.

Characteristic PE bands and degradation-associated functional groups were identified based on their FTIR absorption regions [6,12,13]. The 2919–2851 cm^−1^ region was assigned to the C–H stretching vibrations of the polyethylene backbone, whereas the 1466–1462 cm^−1^ band was associated with CH_2_ deformation. Changes in the 1652–1638 cm^−1^ region were assigned to C=C stretching vibrations associated with unsaturation. The broad 3400–3200 cm^−1^ region was assigned to O–H stretching vibrations of hydroxyl-containing groups, while the 1700–1740 cm^−1^ region was examined for C=O stretching vibrations associated with carbonyl-containing oxidation products. Changes in the 1079–1020 cm^−1^ region were assigned to C–O stretching vibrations and interpreted as oxygen-containing functionalities consistent with alcohol-and/or ether-type structures.

Spectra were baseline-corrected and normalized in the 2800–3000 cm^−1^ region to improve comparability among samples. Three spectroscopic indices were calculated from the integrated absorbance areas: the unsaturation index (UI), calculated as UI = A(1652–1638)/A(1466–1462); the hydroxyl index (HI), calculated as HI = A(3400–3200)/A(1504–1467); and the carbon–oxygen index (COI), calculated as COI = A(1079–1020)/A(2919–2851), where A represents the baseline-corrected integrated area under the absorbance curve within the indicated spectral interval (cm^−1^). The carbonyl region (1700–1740 cm^−1^) was also examined; however, because no well-defined absorption band was detected in the bacteria-treated samples under the in vitro assay conditions, the carbonyl index (CI) was not calculated.

### 2.8. Analysis of Extracellular Proteins by SDS-PAGE Electrophoresis

To identify extracellular proteins potentially responsible for PE-degrading activity, sodium dodecyl sulfate–polyacrylamide gel electrophoresis (SDS-PAGE) was performed on culture supernatants of the three bacterial strains. Bacteria were incubated in CFLBM with PE as the sole carbon source for 7 days at 35 °C under agitation. Extracellular proteins were precipitated with 80% saturation ammonium sulfate, quantified by the Bradford method, denatured with Laemmli buffer (95 °C, 5 min), and separated on a 12% polyacrylamide gel under reducing conditions. Gels were stained with Coomassie Brilliant Blue R-250. SDS-PAGE gel images were analyzed using GelAnalyzer 23.1 software (www.gelanalyzer.com accessed on 31 March 2026). Background correction, lane identification, and automatic band detection were performed. Apparent molecular weights were determined by calibration against a standard protein marker, while relative protein abundance was calculated from the integrated area under each densitometric peak.

### 2.9. Scanning Electron Microscopy (SEM)

Surface morphological alterations of the conventional PE and oxo-degradable PE (OXO) films resulting from Section 2.7 were assessed by scanning electron microscopy (SEM) (Zeiss Evo 40 VP scanning electron microscope, ZEISS Microscopy) ZEISS Group corporate, Jena, Germany, using a secondary electron detector at an accelerating voltage of 7 kV in high-vacuum mode for both inoculated samples and controls. After 45 days of incubation, 5 × 5 mm film fragments were recovered, washed three times with sterile distilled water, dehydrated in an increasing ethanol series (50, 70, 90, and 100%), and dried. Fragments were mounted on aluminum stubs with conductive carbon tape, coated with gold–palladium (~10 nm, sputtering), and observed at 5–15 kV at magnifications of 300×, 500×, and 1000×. Images were generated through an external electron microscopy service in a single capture session per condition on fixed, gold-coated samples; it was therefore not retrospectively possible to generate additional representative fields per biological replicate or to perform quantitative image analysis. SEM images are accordingly presented as qualitative/illustrative evidence of surface colonization and erosion, complementary to the other methods employed.

## 3. Results and Discussion

### 3.1. Molecular Identification of the Bacterial Isolates

16S rRNA gene sequencing allowed the identification of five bacterial isolates from the gut contents of *G. mellonella* larvae subjected to dietary induction with LDPE. In all cases, identity percentages above 99% and E-values of 0.0 were obtained (Table 3).

**Figure 1:**
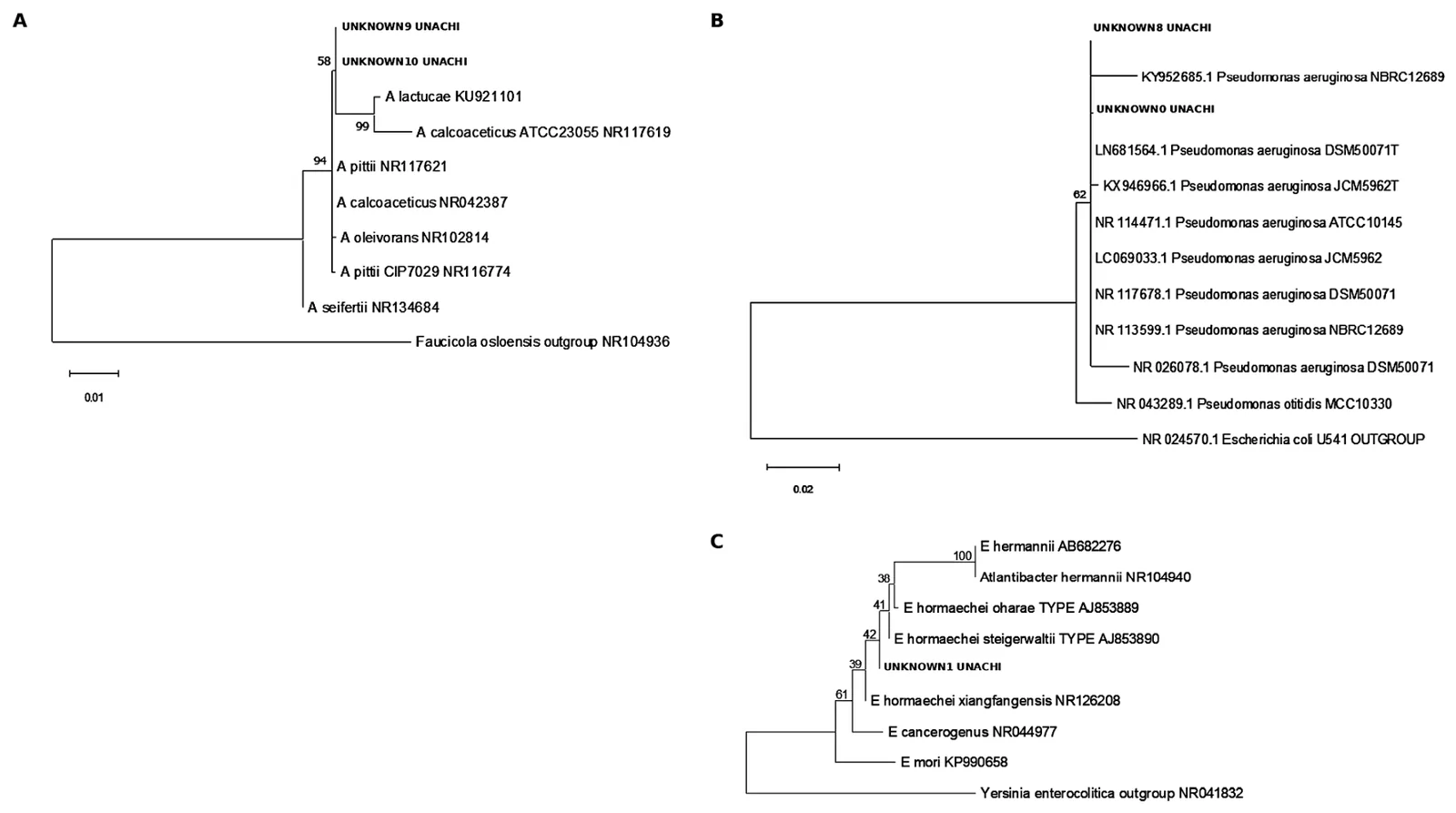
Maximum Likelihood (ML) phylogenetic trees based on 16S rRNA gene sequences of the bacterial isolates obtained from the gut of *Galleria mellonella* larvae. (**A**) Phylogenetic placement of *Acinetobacter pittii* isolates UNKNOWN9 and UNKNOWN10, using *Faucicola osloensis* (NR104936) as the outgroup. (**B**) Phylogenetic placement of *Pseudomonas aeruginosa* isolates UNKNOWN0 and UNKNOWN8, using *Escherichia coli* (NR_026078.1/U541) as the outgroup. (**C**) Phylogenetic placement of *Enterobacter hormaechei* isolate UNKNOWN1, using *Yersinia enterocolitica* (NR041832) as the outgroup. Numbers at the nodes represent bootstrap consensus values (1000 replicates); only values ≥ 30% are displayed. Best-fit nucleotide substitution models were selected using MEGA12 software based on the Bayesian Information Criterion (BIC). Scale bars represent the number of substitutions per site. Note that isolates UNKNOWN9 and UNKNOWN10 cluster within the broader *Acinetobacter calcoaceticus–baumannii* (ACB) complex rather than forming an exclusive clade with *A. pittii* reference sequences, reflecting the well-documented limited resolving power of the 16S rRNA gene within this species complex.

The five isolates correspond to three species: *Pseudomonas aeruginosa* (UNKNOWN0 and UNKNOWN8), *Enterobacter hormaechei* (UNKNOWN1), and *Acinetobacter pittii* (UNKNOWN9 and UNKNOWN10). The presence of *P. aeruginosa* is consistent with previous reports [4,5], confirming it as a positive biological reference. The prevalence of *Pseudomonas aeruginosa* (isolates UNKNOWN0 and UNKNOWN8) in the gut microbiome of LDPE-fed larvae is a widely validated finding in the literature, where this species is described as one of the most effective microorganisms for polyethylene biodegradation (Table 3; Figure 1). Previous studies have shown that *P. aeruginosa* not only survives in minimal media with plastic but actively proliferates using PE as the sole carbon source. Its catabolic capacity is underpinned by key enzymatic systems, such as alkane monooxygenase (alkB1 and alkB2 genes), which initiate oxidation of the polymer’s carbon chains [4,14].

In contrast, *E. hormaechei* and *A. pittii* represent the most novel findings: the literature contains only a few reports of PE-degrading activity documented individually for these two species [15]. Isolation from a microbiome enriched by three months of dietary selective pressure with PE suggests that in vivo metabolic induction is an effective mechanism for identifying strains with catabolic activity toward synthetic polymers, consistent with the concept of the insect as a selection bioreactor [1].

The identification of *Enterobacter hormaechei* (isolate UNKNOWN1) also provides relevant information on the diversity of the genus Enterobacter in plastic degradation. While species such as *E. asburiae* and *Enterobacter* sp. have previously been isolated from the guts of lepidopteran larvae with documented activity toward PE, *E. hormaechei* has recently been reported in isolation studies using polyethylene as the sole energy source, suggesting a similar adaptive capacity within the genus [16].

The finding of *A. pittii* (isolates UNKNOWN9 and UNKNOWN10) is particularly notable. It should be noted that species within the *Acinetobacter calcoaceticus–baumannii* (ACB) complex, including *A. calcoaceticus*, *A. pittii*, *A. baumannii*, *A. nosocomialis*, *and A. seifertii*, are frequently difficult to resolve using the 16S rRNA gene alone due to its high sequence conservation within this group. Accordingly, the identification of UNKNOWN9 and UNKNOWN10 as *A. pittii*, while supported by top BLASTn hits (99.86% identity), should be interpreted with appropriate caution pending confirmation by higher-resolution methods (e.g., multilocus sequence analysis or whole-genome average nucleotide identity). Although recent studies have highlighted *Acinetobacter guillouiae* as a novel PE degrader, metagenomic investigations indicate that *A. pittii* possesses the metabolic pathways necessary to use non-pretreated LDPE as a substrate for growth [15]. Bacteria of the genus Acinetobacter are known for their ability to degrade hydrocarbons due to the structural similarity between these compounds and lipids, which facilitates their colonization of the plastic surface [17].

The successful isolation of these strains after three months of dietary induction reinforces the theory of the insect as a selection bioreactor. This prolonged period of selective pressure allows for the differential enrichment of microorganisms that not only possess the necessary genes but also maintain active enzymatic machinery (oxidoreductases, hydrolases) required to fragment and assimilate synthetic polymers. No positive results were obtained in colorimetric tests when these bacteria were isolated from the control group, without prior contact with plastic; as discussed above, this underscores that in vivo induction is an essential methodological step to activate their latent metabolic capabilities [15].

### 3.2. Metabolic Activity Toward PE: DCPIP Assay

DCPIP reduction percentages reached maximum values of 96.07% (*P. aeruginosa*), 95.93% (*E. hormaechei*), and 93.80% (*A. pittii*) on day 7. The difference between the bacterial strains and Control 2 was statistically significant (Kruskal–Wallis H = 10.71; *p* = 0.013). Control 1 (abiotic) maintained a constant absorbance of 3.000, confirming the stability of the indicator.

**Figure 2:**
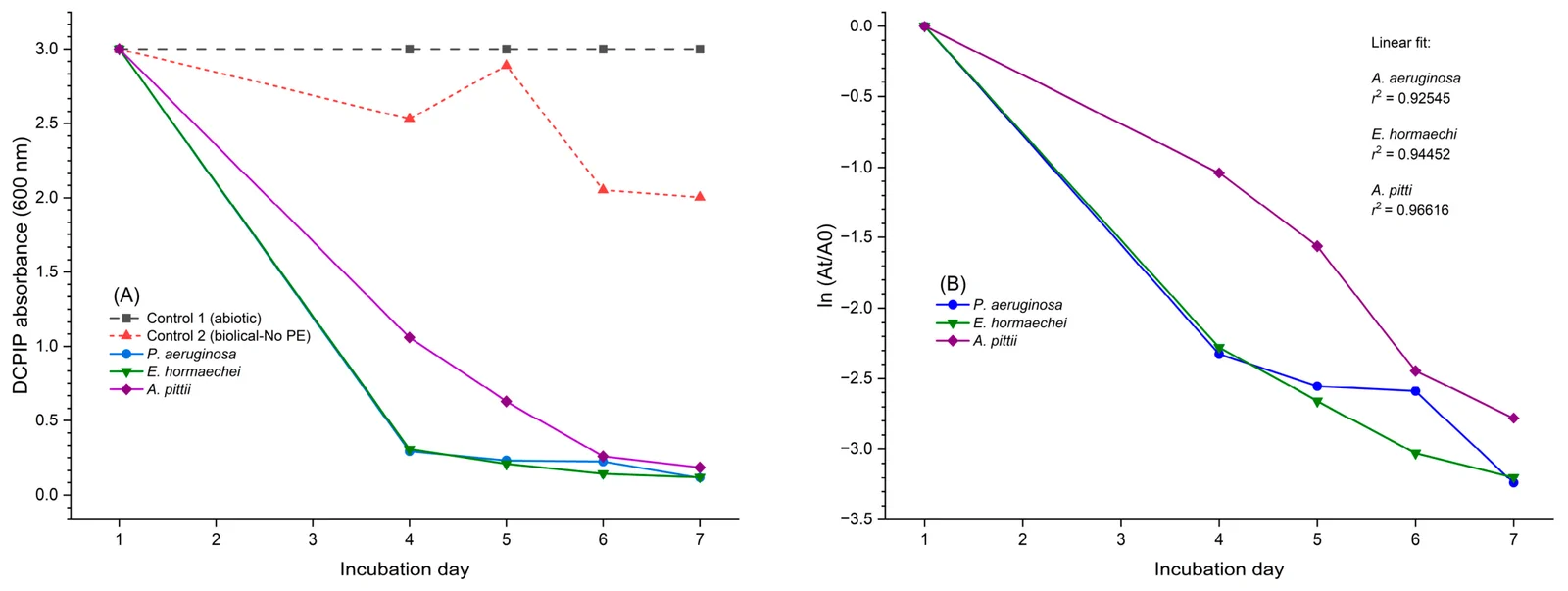
(**A**) Kinetics of DCPIP redox indicator absorbance (600 nm) over 7 days of incubation with PE as the sole carbon source. Control 1: abiotic control (DCPIP + PE, no bacteria); Control 2: biological control (DCPIP + bacteria, no PE). All three bacterial strains showed a drastic reduction from day 4, indicating polymer-mediated oxidative activity. (**B**) First-order kinetics analysis of DCPIP reduction. ln (At/A0) is plotted against incubation day with linear regressions for each strain. Equivalent slopes (*p* > 0.97) indicate parallel first-order kinetics across all three species.

First-order kinetic analysis revealed apparent rate constants of k = −0.5198 day^−1^ (*P. aeruginosa*), k = −0.5483 day^−1^ (*E. hormaechei*), and k = −0.4766 day^−1^ (*A. pittii*), with R^2^ between 0.92 and 0.97 and half-reduction times of 1.26–1.45 days. The interaction model confirmed parallel kinetics among the three species (*p* > 0.97; Figure 2B).

The marked increase in the percentage of DCPIP reduction from day 4 onward (Figure 2A) suggests that all three isolates (*P. aeruginosa*, *E. hormaechei*, and *A. pittii*) possess the enzymatic machinery necessary to oxidize the hydrocarbon chains of LDPE. The maximum values reached on day 7, exceeding 93% in all species (Table 4), are consistent with studies proposing this period as the ideal window for identifying bacteria with high degradative efficiency using this redox indicator [11]. The stability of Control 1 (abiotic) ensures that the observed decolorization is a strictly biological process rather than a spontaneous chemical reduction of the indicator by the polymer (Table 4).

The first-order kinetics, with similar apparent rate constants (Figure 2B) and half-reduction times between 1.26 and 1.45 days, could indicate a similar oxidation mechanism among the three species. This behavior supports the theory that polyethylene biodegradation follows a conserved pathway that begins with alkane hydroxylation and alcohol dehydrogenation, key steps in converting the polymer into assimilable products such as fatty acids. The fact that *P. aeruginosa* and *A. pittii* exhibit comparable kinetics reinforces previous findings in which bacteria of the genus Acinetobacter have shown proliferation and PE-utilization rates comparable to those of well-known degraders such as Pseudomonas [18].

It is notable that the metabolic induction achieved through partial dietary replacement over three months in the larvae is a determining factor for this rapid response. In strains isolated from the control group, and in the literature, it has been observed that strains isolated without this prior selective pressure often do not show immediate activity in colorimetric tests, which highlights that prolonged in vivo exposure activates latent enzymatic pathways, such as the alkane monooxygenase (alkB) systems [4]. The ability of these isolates to reduce DCPIP so efficiently positions this consortium as a promising agent for biofragmentation and bioassimilation processes of recalcitrant plastics [19].

### 3.3. Bacterial Metabolic Resistance Test

#### 3.3.1. *Pseudomonas aeruginosa*

The two-way repeated measures ANOVA with replicates (n = 2) on experimentally measured days (1, 3, and 5) revealed that both physical stress treatments (F = 806.72; *p* < 0.0001) and incubation time (F = 1953.39; *p* < 0.0001) had highly significant main effects on relative PE consumption, with a very strong treatment × time interaction (F = 236.88; *p* < 0.0001) (Table S4—ANOVA). Post hoc pairwise comparisons (Fisher’s LSD, α = 0.05) on day 5 showed a significant decrease in relative consumption under physical stress, with values reaching 73.83% (normal), 43.17% (UV), and 34.17% (boiling). The marked post-boiling reduction suggests partial enzymatic denaturation, whereas the post-UV persistence indicates robust residual functionality under moderate irradiation. This capability of the bacterial machinery to keep functioning after severe thermal shock suggests that the degradative enzymes of *P. aeruginosa* possess structural features that prevent complete denaturation, or exhibit a rapid renaturation capacity.

#### 3.3.2. *Enterobacter hormaechei*

For *E. hormaechei*, the corrected two-way ANOVA confirmed highly significant main effects for both the physical treatments (F = 255.40; *p* < 0.0001) and incubation time (F = 2555.54; *p* < 0.0001), as well as a highly significant treatment × time interaction (F = 121.85; *p* < 0.0001) (Table S4—ANOVA). Notably, on day 5, relative consumption under UVC + O_3_ treatment (61.28%) slightly exceeded the normal treatment (57.95%). This difference, although moderate, suggests potential enzymatic induction triggered by oxidative stress. This finding, novel for *E. hormaechei* with respect to synthetic polymers, is highly consistent with the overexpression of inducible oxidases, laccases, or peroxidases to mitigate oxidative damage [20].

#### 3.3.3. *Acinetobacter pittii*

*A. pittii* proved to be the most metabolically versatile strain, exhibiting highly significant main effects for physical treatments (F = 178.46; *p* < 0.0001) and incubation time (F = 4335.32; *p* < 0.0001), along with a highly significant interaction factor (F = 103.62; *p* < 0.0001) (Table S4—ANOVA). Post hoc comparisons revealed that this strain reached its highest degradative acceleration under UVC + O_3_ on day 3 with 77.13% relative consumption, significantly outperforming the normal condition (63.70%; Fisher’s LSD, *p* < 0.001). Furthermore, its post-boiling activity on day 5 (50.88%) was the highest among the three strains. This extraordinary thermotolerance suggests the presence of specialized thermoresistant isoenzymes capable of maintaining catalytic activity or rapidly renaturing after thermal shock.

A distinctive aspect of this research is the exposure of the bacterial isolates to extreme physical stress conditions (boiling and UV radiation). While most biodegradation studies are conducted under laboratory conditions optimized for microbial growth (generally between 25 °C and 37 °C), in natural ecosystems plastics are exposed to weathering processes that include cycles of high ultraviolet radiation and severe thermal fluctuations. By evaluating the metabolic capacity of the bacteria after these treatments, this experiment measures not only their catabolic potential but also the validation and robustness of the consortium for real-world bioaugmentation applications in hostile environments.

#### 3.3.4. Validation Under Thermal Shock

Boiling treatment (100 °C for 15 min) acts as a confirmatory test of the enzymatic nature of the degradation process. In microbiology, this procedure is the standard method for inducing thermal denaturation of proteins and the consequent inactivation of biocatalysts [21]. The significant reduction in LDPE consumption observed in all three strains after boiling (especially in *P. aeruginosa*, which dropped from 73.83% to 34.17%) confirms that degradation is mediated by heat-sensitive biological agents (enzymes) rather than by a residual chemical oxidation phenomenon [22].

However, the residual functionality detected in *Acinetobacter pittii* (50.88%) after boiling is a finding of high biotechnological value. This resistance suggests the possible presence of thermotolerant isoenzymes or a capacity for rapid protein renaturation; characteristics reported in bacteria that inhabit niches with recurrent thermal stress. This thermal resilience positions *A. pittii* as an important species for bacterial consortia intended for industrial composting or bioremediation processes in extreme climates [23].

#### 3.3.5. Validation Under UV Radiation

The response of the isolates to UV radiation reinforces the theory of synergy between abiotic and biotic factors in the breakdown of recalcitrant polymers [19]. In the environment, UV rays act as a primary abiotic deterioration agent, inducing the formation of carbonyl groups (–C=O–) and free radicals on the plastic surface, which facilitates its subsequent microbial assimilation [24].

The finding that *Enterobacter hormaechei* (61.28%) and *Acinetobacter pittii* (77.13%) maintained or even exceeded their normal consumption levels after UV irradiation suggests the presence of an enzymatic induction mechanism triggered by oxidative stress [15,23]. Rather than being inhibited by radiation, these strains appear to possess regulatory systems that activate the overexpression of oxidoreductases or peroxidases to mitigate oxidative damage. These enzymes are, in turn, essential for initiating the oxidative attack on the polyethylene carbon backbone [15]. This marked metabolic responsiveness allows the consortium not only to tolerate the environmental stress that favors plastic degradation but also to exploit it to enhance its catabolic machinery.

#### 3.3.6. The Insect as Bioreactor and Its Biotechnological Relevance

The fact that these strains retain high activity after physical stress processes validates the prolonged dietary enrichment method used in *G. mellonella* larvae. Subjecting the microbiome to a plastic-based diet for three months made it possible to select not only the most effective degraders but also the most robust ones [25]. This approach is consistent with the view of the larva as a selection bioreactor, in which in vivo selective pressure activates latent metabolic pathways that would otherwise remain inactive in conventional culture media. The demonstrated ability of all three species to function under adverse light and temperature conditions makes them ideal candidates for mitigating plastic pollution under field conditions, where organisms must survive the rigors of direct solar exposure and constant thermal fluctuations.

### 3.4. Gravimetric Assessment and Daily Degradation Rate

*A. pittii* exhibited the greatest degradation of conventional PE (3.13%), followed by *E. hormaechei* (2.52%) and *P. aeruginosa* (1.29%). The gravimetric assay was performed with n = 2 biological replicates per strain (each measured in technical triplicate) against a control of n = 3. Applying Welch’s *t*-test to the biological-replicate means, none of the three strains reached formal statistical significance on PE under this corrected design: *E. hormaechei* (*t*-test = 4.623; *p* = 0.136), *A. pittii* (*t*-test = 2.845; *p* = 0.215), and *P. aeruginosa* (*t*-test = 1.652; *p* = 0.347) (Table 5). Given the limited statistical power inherent to n = 2, these results are presented as a consistent, biologically coherent trend of mass loss across strains rather than as a statistically significant outcome.

On OXO, applying the same corrected Welch’s *t*-test design (n = 2 biological replicates vs. control n = 3), *A. pittii* showed a statistically significant mass loss (t = 6.928; *p* = 0.011), while *E. hormaechei* (t = 2.738; *p* = 0.191) and *P. aeruginosa* (t = 0.663; *p* = 0.593) did not reach significance. The lower degradation observed on oxo-degradable films may be attributed to the absence of the abiotic pretreatment needed to activate the pro-oxidant additives, and is consistent with previous studies reporting higher biodegradation values for non-pretreated PE than for non-pretreated oxo-degradable plastic bags [8,26].

As previously noted, all strains were exposed to native PE without abiotic pretreatment, unlike most published studies [2,4], suggesting that these strains are of particular biotechnological relevance. *E. hormaechei* and *A. pittii* apparently possess the enzymatic machinery needed to initiate degradation of the polymer under real environmental conditions, validating the efficacy of the in vivo induction protocol as a functional enrichment strategy.

The mass loss observed in conventional polyethylene (PE) films, which reached a maximum of 3.13% for *A. pittii* and 2.52% for *E. hormaechei* (Table 5), is a solid indicator of genuine biodegradation. In the literature, Acinetobacter strains have been reported to show mass-reduction capacities comparable to those of well-known degraders such as *Pseudomonas aeruginosa*, achieving assimilation of the polymer as the sole carbon and energy source [15]. The daily degradation rate of up to 0.696 mg/day for *A. pittii* positions this strain as a highly efficient agent, considering that biodegradation of plastics with a carbon–carbon backbone is an intrinsically slow process under natural conditions [5].

While it is true that the biodegradation rates reported for these three strains under the assay conditions appear lower than those found for other species in the literature, it is also important to highlight that most documented studies use polymers subjected to prior UV radiation or thermal treatments to induce initial oxidation (“hydroperoxidation”) that facilitates bacterial attack [19].

The fact that the isolates achieved a consistent trend of mass loss on pristine films suggests that they possess highly specialized metabolic tools capable of autonomously initiating polymer deterioration and fragmentation [24].

This finding validates the efficacy of the three-month in vivo induction protocol, which acted as a mechanism for selecting strains with robust and active catabolic capacities, reinforcing the view of the *G. mellonella* gut as a functional selection bioreactor [27]. Regarding the oxo-degradable polyethylene (OXO) films, the lower degradation observed (maximum 1.37% for *A. pittii*) is consistent with reports indicating that the pro-oxidant additives present in OXO plastics (such as transition-metal salts) require thermal or light energy to catalyze the breakdown of polymer chains into assimilable fragments. Without this abiotic “trigger,” the material retains its recalcitrant structure, limiting bacterial bioassimilation. Nevertheless, the ability of *A. pittii* to generate a statistically significant mass loss even under these OXO conditions, together with the consistent (though not statistically significant) trend observed for *E. hormaechei*, underscores the metabolic versatility of these strains toward different synthetic polymer formulations.

### 3.5. Comparison Between Methodologies for Quantifying Degradative Activity

**Figure 3:**
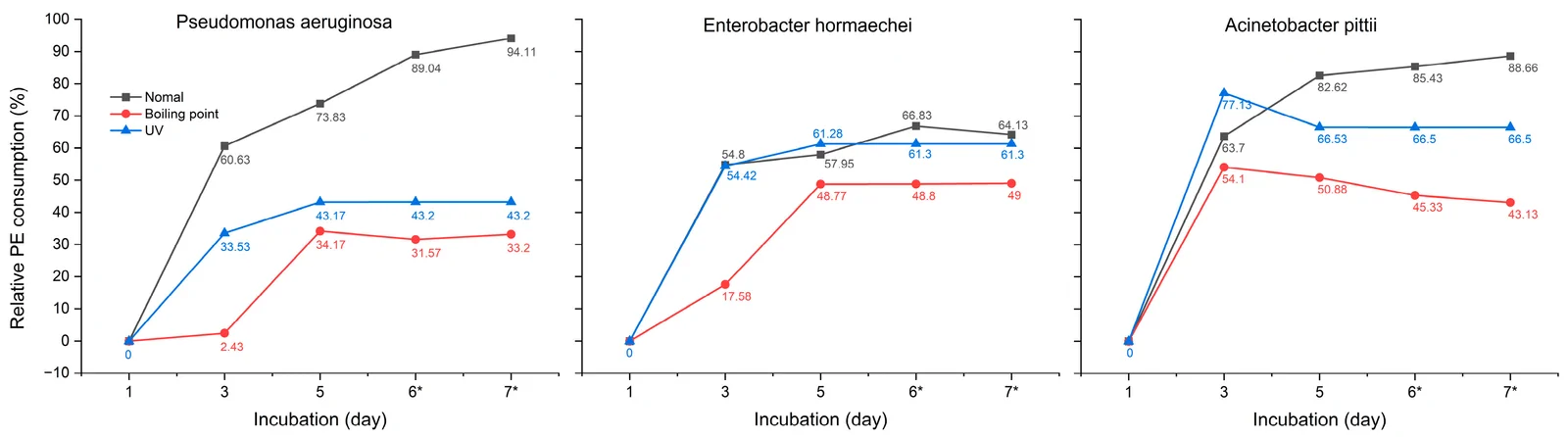
Relative PE consumption percentage under three physical stress treatments (normal, boiling at 100 °C/15 min, UV/15 min) for the three bacterial strains at days 1, 3, 5, 6, and 7. *P. aeruginosa*, *E. hormaechei,* and *A. pittii* showed UV activity equal to or higher than the normal treatment, indicating possible enzyme induction by oxidative stress. Asterisks (*) at days 6 and 7 denote estimated values projected by trend extrapolation for boiling and UV curves. Error bars and statistical comparisons are strictly based on experimental data from days 1, 3, and 5.

Figure 3 presents a direct comparison between the metabolic activity quantified by the DCPIP assay at day 7 and the cumulative gravimetric mass loss at 45 days for the three strains on conventional PE and OXO. All three strains converged on high and mutually similar DCPIP consumption values (93.8–96.1% at day 7), without marked differentiation between species. In contrast, gravimetric mass loss showed greater discrimination among strains, with *A. pittii* reaching the greatest reduction in conventional PE (3.13%, *p* = 0.0215), followed by *E. hormaechei* (2.52%, *p* = 0.136) and *P. aeruginosa* (1.29%, *p* = 0.347)—notably, the ranking of strains by mass loss was the inverse of that observed for DCPIP consumption. Given the small number of strains evaluated (n = 3), a formal correlation analysis between the two variables was not performed; however, the observed pattern does not suggest a direct proportional relationship between the two assays. This is consistent with the nature of each method: DCPIP reflects general respiratory/redox activity of the strain toward the polymer as a carbon source, while gravimetry quantifies net cumulative mass loss over the long term, an outcome that also depends on other factors (cell adherence, biofilm formation, extracellular enzymatic activity sustained over time). Both assays should therefore be interpreted as complementary, rather than equivalent, lines of evidence [11]. The difference in time scale between the two methods—7 days for DCPIP and 45 days for gravimetry—reflects that each captures complementary aspects of the degradation process: initial oxidative activity kinetics versus net cumulative mass loss.

The high efficacy of *A. pittii* on both metrics reinforces the biotechnological relevance of this genus, which has recently been described as possessing enzymatic biodegradation capabilities (including possible alkane monooxygenases and esterases) able to degrade PE comparably to, or even better than, classical degraders such as Pseudomonas. Finally, this methodological comparison underscores that the combined use of short-term colorimetric screening techniques and long-term physical assessments is the most rigorous strategy for characterizing the full biodegradation cycle of recalcitrant plastics, allowing differentiation between the capacity to “activate” the polymer and the efficiency of converting it into biomass [28].

### 3.6. Structural Characterization of PE and OXO Films by FTIR-ATR

No defined band was detected in the 1700–1740 cm^−1^ region (C=O) in bacteria-treated samples, indicating the absence of deep carbonyl oxidation in vitro (Table 6). A notable spectroscopic feature, however, was the presence of a weak carbonyl band at 1718 cm^−1^ in the pristine, untreated commercial PE powder (PE 500 mesh). This pre-existing oxidation is attributed to two factors: (i) the localized thermal and mechanical stress induced during the industrial micronization process required to achieve the 500-mesh particle size, which facilitates superficial thermo-oxidation, and (ii) the potential presence of carbonyl-containing thermo-stabilizers added during manufacturing. It is worth noting that while commercial library matching often assigns a high similarity score to high-density polyethylene (T-HDPE) due to the identical carbon–carbon covalent backbone structure, ATR-FTIR spectroscopy possesses limited resolution to physically discriminate polymer densities (LDPE vs. HDPE) without complementary density or thermal assessments.

Under in vitro incubation, the bacteria rapidly colonize the polymer surface and establish a mature biofilm. We hypothesize that these pre-existing, easily accessible oxidized carbonyl groups on the powder surface are preferentially targeted and consumed by the bacterial consortium as a readily assimilable carbon source during early colonization. This rapid bio-assimilation of pre-oxidized fragments accounts for the disappearance of the 1718 cm^−1^ band in the bacteria-treated films, which falls below the spectrophotometric detection limit. This metabolic preference explains why the in vitro degradative process proceeds via an alternative, hydroxyl- and ether-dominated pathway (AlkB-mediated), without accumulating detectable carbonyl intermediates in the remaining polymer matrix [15].

This absence of newly accumulated carbonyl groups in vitro contrasts with the deep carbonylic oxidation reported in in vivo studies with *Galleria mellonella* [8]. This discrepancy is consistent with attributing the carbonylic oxidation observed in vivo primarily to host enzymes and mechanical processes [7], suggesting that polyethylene degradation in the insect is a compartmentalized and synergistic process [25]. In the complete biological system, initial oxidation and carbonyl group formation are primarily driven by the mechanical action of mastication together with the secretion of specialized enzymes in the larval saliva, such as arylphorins and hexamerins, which act as a biotic pretreatment that weakens the polymer structure [29].

In the absence of these host enzymes, our bacterial isolates may operate via a proposed complementary metabolic mechanism: the alkane pathway, suggested by surface modification through the incorporation of hydroxyl groups (HI = 0.68–1.25 in OXO) and C–O-containing functionalities (variable COI) (Table 6), pointing to activation of the alkane monooxygenase (AlkB) system [15] and subsequent β-oxidation of alcohol intermediates. Once primary alcohols are formed via AlkB-mediated terminal hydroxylation, they would plausibly be rapidly converted by bacterial dehydrogenases into aldehydes and fatty acids destined for cellular mineralization, which prevents their detectable accumulation as carbonyl intermediates on the polymer surface.

Under this model, the first biotic step is likely hydroxylation of the carbon chain to form alcohols. The lack of carbonyl group accumulation on the plastic surface in vitro may be explained by a superior catabolic efficiency of the isolated strains; once formed, alcohols are rapidly converted into aldehydes and fatty acids that enter β-oxidation and the Krebs cycle for mineralization, avoiding their detectable persistence via surface spectroscopy of the film. Finally, these results reinforce the hypothesis that biodegradation of recalcitrant plastics is significantly more efficient when the combined action of the insect and its microbiota occurs.

While the larva’s enzymes initiate biodeterioration through aggressive oxidation that generates carbonylated microplastics, the bacterial consortium acts as the engine of final bioassimilation via the alkane pathway. It is highly likely that this synergistic interaction is responsible for the high rates of mass degradation observed in the living insect, which exceed in speed and magnitude the capabilities of the bacteria operating individually in conventional culture media [19]. This functional complementarity positions the in vivo-enriched microbiome as a key biotechnological tool for processes requiring rapid mineralization of synthetic polymers.

### 3.7. Analysis of Extracellular Proteins by SDS-PAGE Electrophoresis

**Figure 4:**
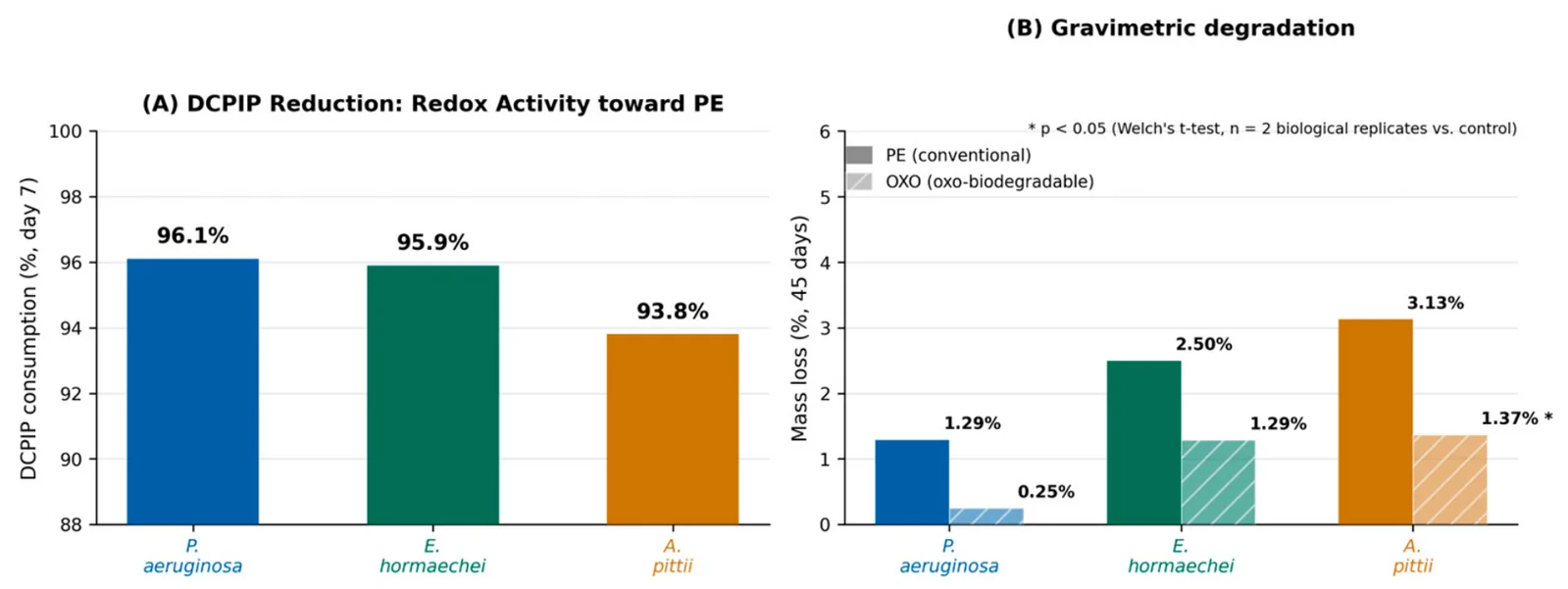
Comparison between DCPIP-based metabolic activity (day 7, %) and gravimetric mass loss (45 days, %) for PE conventional films (solid bars) and OXO oxo-biodegradable films (hatched bars). (**A**) DCPIP consumption (%) at day 7. (**B**) Gravimetric mass loss (%) for PE and OXO films, labeled above each bar; * *p* < 0.05 (Welch’s *t*-test, n = 2 biological replicates vs. control n = 3)—only *A. pittii* on OXO reaches statistical significance under this corrected design. Colors: blue = *P. aeruginosa*; green = *E. hormaechei*; amber = *A. pittii.*

**Figure 5:**
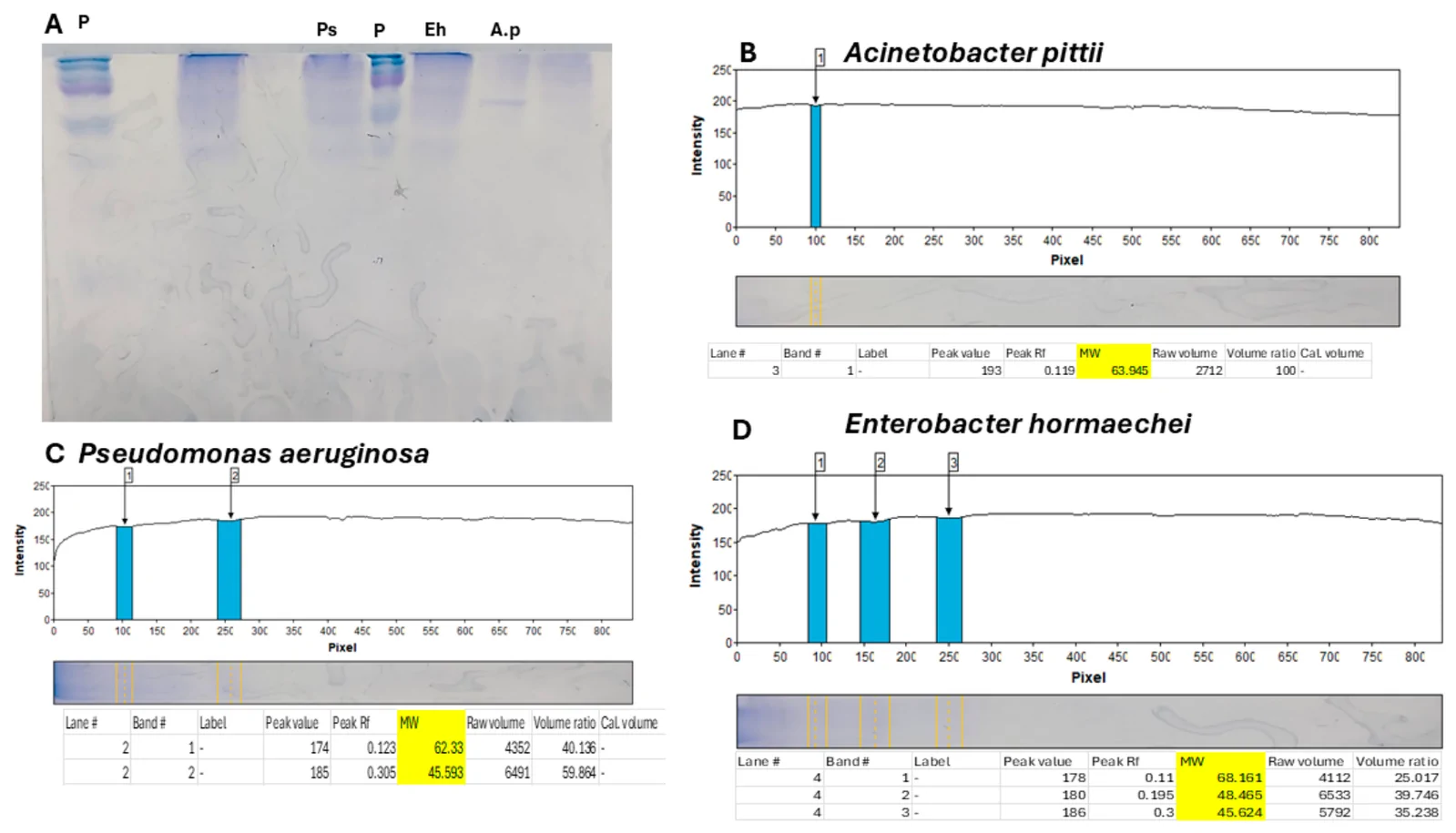
SDS-PAGE analysis of unpurified total protein extracts from the three polyethylene (PE)-degrading bacterial isolates studied in this work. (**A**) Original gel photograph (contrast-enhanced only), showing the electrophoretic separation of the protein extracts from all three strains. The dotted line separates two loading volumes of the same samples: left lanes, lower loading volume; right lanes, higher loading volume, giving more clearly visible bands. (**B**) Densitometric analysis of the *Acinetobacter* sp. (closely related to *A. pittii*) lane, performed with GelAnalyzer software. The intensity-versus-pixel profile (top) shows one resolved peak, marked by a numbered box (Band #1) that corresponds to the “Band #” column of the output table below; the lane image used for the analysis is shown beneath the profile; the blue-shaded area under the peak represents the integrated peak area used by the software to calculate the relative band intensity (“Raw volume” and “Volume ratio” columns). The output table (bottom) lists this band’s peak value, peak Rf, interpolated molecular weight (63.95 kDa, highlighted), raw volume and volume ratio. (**C**) Densitometric analysis of the *Pseudomonas aeruginosa* lane, following the same layout as (**B**). Two resolved bands were detected: Band #1 (62.33 kDa) and Band #2 (45.59 kDa). (**D**) Densitometric analysis of the *Enterobacter* sp. (closely related to *E. hormaechei*) lane, following the same layout as (**B**). Three resolved bands were detected: Band #1 (68.16 kDa), Band #2 (48.47 kDa) and Band #3 (45.62 kDa). Putative enzymatic identities for these molecular weight ranges, based on PE-degrading enzymes reported for the same genera (AlkB and laccase/multicopper oxidase, MCO), are discussed in Section 3.6; putative identity pending biochemical/molecular confirmation.

SDS-PAGE analysis of concentrated, unpurified total protein extracts revealed faint but detectable bands in all three bacterial strains evaluated (Figure 4). For *Pseudomonas* sp., two bands were observed at 62.3 kDa and 45.6 kDa (volume ratio 40:60). *Acinetobacter* sp. showed a single band at ~64.0 kDa. *Enterobacter* sp. showed three bands at ~68.2, ~48.5, and ~45.6 kDa, detectable only at the higher loading volume; no bands were recovered at the lower volume, consistent with low protein yield under non-inductive conditions. Molecular weights were calculated by interpolation from a six-band marker (37–250 kDa; R^2^ = 0.993) (Figure 5).

#### Putative Enzymatic Identity of the Detected Bands

The molecular weights of the detected bands are consistent with those of key enzymes implicated in polyethylene (PE) biodegradation reported for the same bacterial genera. In *Pseudomonas aeruginosa*, the ~45.6 kDa band falls within the mass range of alkyl monooxygenase (AlkB; ~41–44 kDa), the main enzyme catalyzing terminal hydroxylation of PE’s alkane chains, whose central role in the degradation of low-molecular-weight PE was confirmed through heterologous expression and carbon mineralization assays [4,14]. The ~62–69 kDa band could correspond to a laccase-type multicopper oxidase (MCO), whose reported masses in Pseudomonas range between 53 and 75 kDa [30]. In *Acinetobacter pittii*, the ~64 kDa band closely matches the 69 kDa reported for an MCO of this genus—a laccase-type MCO from *A. baumannii* Rd-H2 confirmed by SDS-PAGE after heterologous expression, with demonstrated capacity to directly degrade PE film, with laccase activity of up to 159.82 U/L [31]; a structurally related laccase of similar mass was recently reported in *A. dijkshoorniae* [32]. In *Enterobacter* sp., the ~68 kDa band is compatible with the 75 kDa thermo-acid-tolerant laccase characterized in *Enterobacter hormaechei* AI1 [33], while the ~45–48 kDa bands fall within the mass range of the alkyl monooxygenase identified in E. cloacae O5-E [34]. The set of samples with higher loading volume produced more intense and additional bands, consistent with the low constitutive expression expected of these enzymes under non-inductive conditions [5]. Given the absence of purification, the identity of the bands remains hypothetical; confirmation by Western blot or mass spectrometry (LC-MS/MS) is required for a definitive assignment. Nevertheless, the presence of protein bands suggests the production of functional extracellular molecules that could have metabolic roles.

### 3.8. Morphological Analysis by Scanning Electron Microscopy (SEM)

Morphological analysis by SEM revealed characteristic surface alterations in PE and OXO films incubated with the three strains, in contrast to the smooth surface of the abiotic controls.

#### 3.8.1. *Acinetobacter pittii*

**Figure 6:**
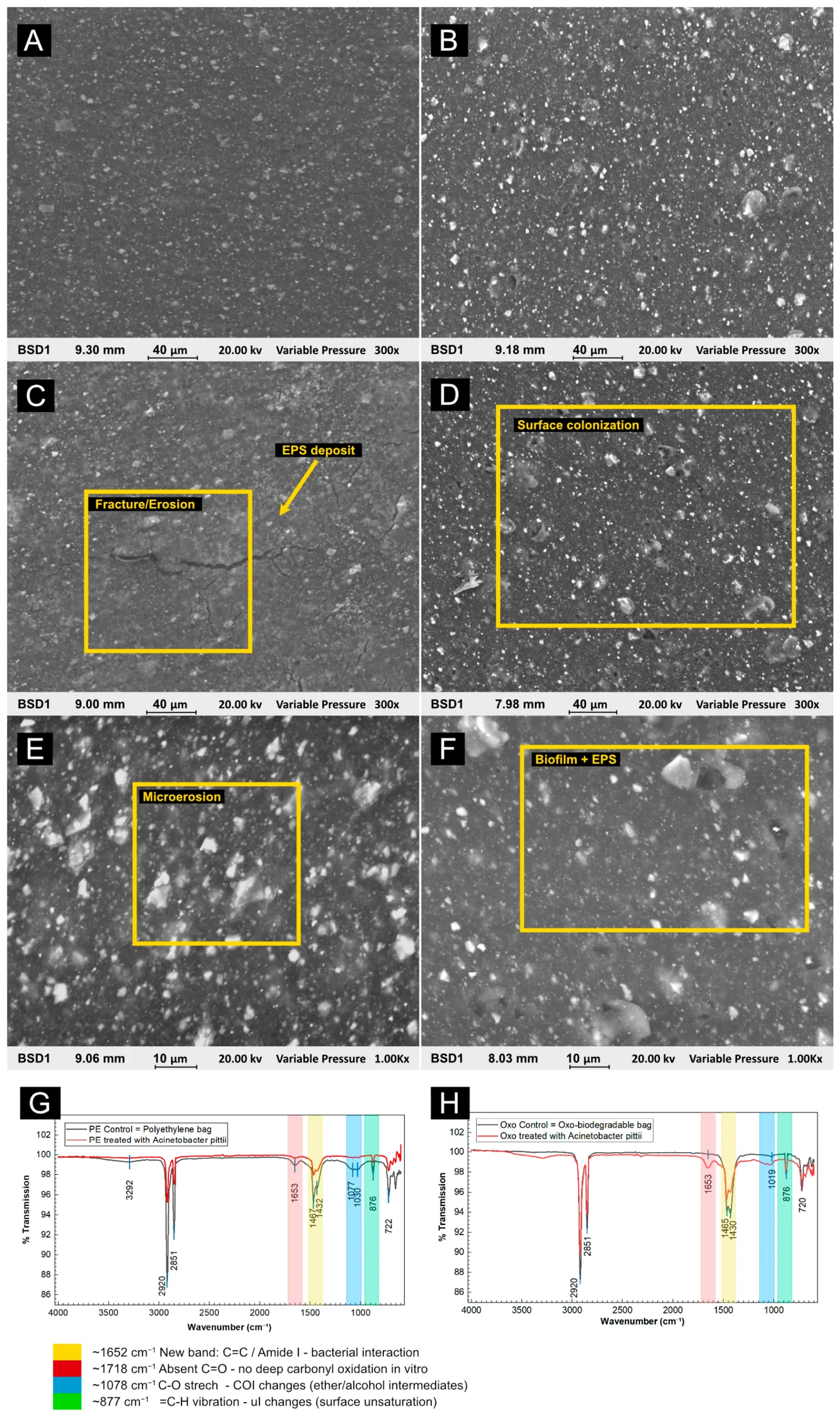
SEM micrographs and FTIR-ATR spectra of PE (left) and OXO (right) films treated with *Acinetobacter pittii* after 45 days of incubation, compared with the abiotic controls ((**A**): PE control; (**B**): OXO control). (**C**) Surface alterations of PE at 300×, showing fracture/erosion and an associated EPS (extracellular polymeric substance) deposit. (**D**) Surface alterations of OXO at 300×, showing surface colonization by bacterial cells and aggregates. (**E**) Surface alterations of PE at 1000×, showing microerosion. (**F**) Surface alterations of OXO at 1000×, showing biofilm formation together with EPS. (**G**,**H**) FTIR-ATR spectra of PE (**G**) and OXO (**H**) showing unsaturation index (UI) and hydroxyl index (HI) values.

Micrographs showed bacterial biofilm with EPS deposition, microstructures, and localized erosion at 500× and 1000×. OXO films exhibited greater susceptibility to colonization, consistent with the higher HI (0.68–1.25) and greater gravimetric degradation (3.13%) recorded *for A. pittii* (Figure 6).

#### 3.8.2. *Enterobacter hormaechei*

**Figure 7:**
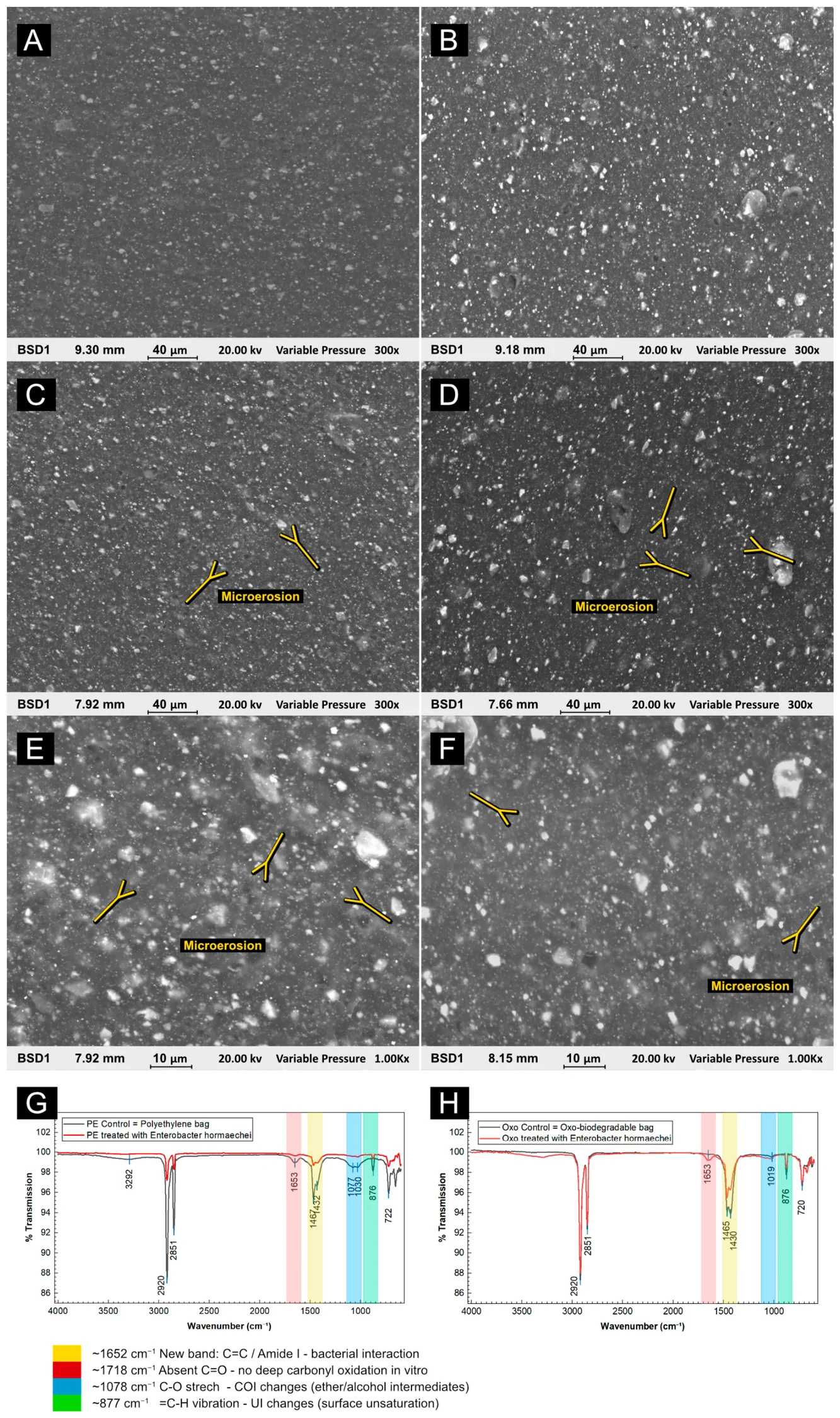
SEM micrographs and FTIR-ATR spectra of PE (left) and OXO (right) films treated with Enterobacter hormaechei after 45 days of incubation, compared with the abiotic controls ((**A**): PE control; (**B**): OXO control). (**C**,**D**) Surface alterations of PE (**C**) and OXO (**D**) at 300×, showing microerosion. (**E**,**F**) Surface alterations of PE (**E**) and OXO (**F**) at 1000×, showing microerosion. (**G**,**H**) FTIR-ATR spectra of PE (**G**) and OXO (**H**), showing reduced spectral scores compared to the control, reflecting detectable structural modifications.

EPS deposits and localized microerosion zones were observed at 500× and 1000×, evidence of extracellular enzymatic action. FTIR-ATR scores dropped to 836–848 versus 871 for the control, reflecting detectable structural modifications. Mass loss: 2.50% on PE (*p* = 0.136) (Figure 7).

#### 3.8.3. *Pseudomonas aeruginosa*

**Figure 8:**
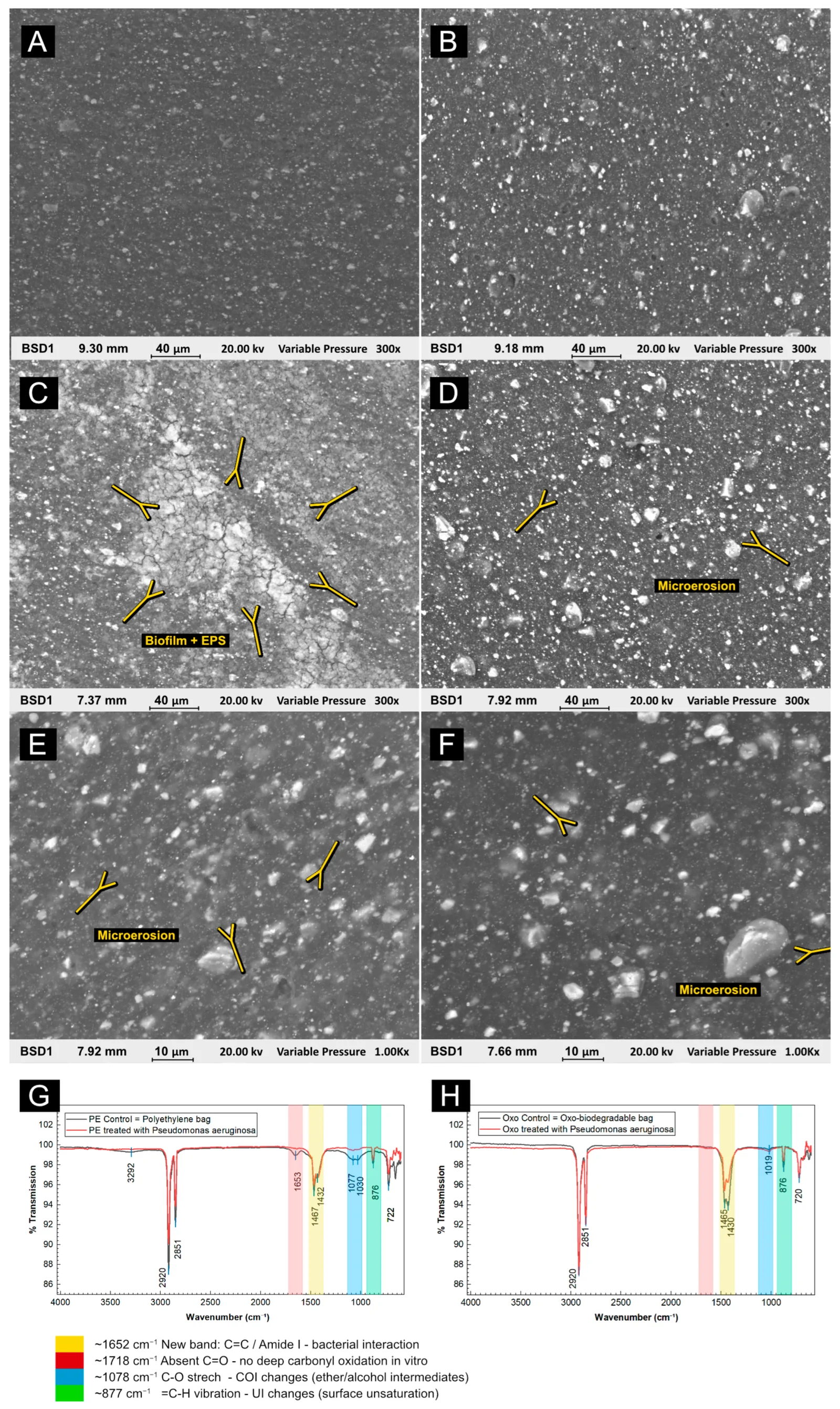
SEM micrographs and FTIR-ATR spectra of PE (left) and OXO (right) films treated with *Pseudomonas aeruginosa* after 45 days of incubation, compared with the abiotic controls ((**A**): PE control; (**B**): OXO control). (**C**) Surface alterations of PE at 300×, showing biofilm formation together with EPS (extracellular polymeric substance) deposits. (**E**) Surface alterations of PE at 1000×, showing microerosion. (**D**,**F**) Surface alterations of OXO at 300× and 1000×, respectively, showing microerosion. (**G**,**H**) FTIR-ATR spectra of PE (**G**) and OXO (**H**) showing unsaturation index (UI) and hydroxyl index (HI) values.

Structured biofilms with EPS channels characteristic of *P. aeruginosa* formed. PE surfaces exhibited grooves and linear discontinuities at 1000×. SEM data confirm direct physical interaction between bacterial cells and the polymer surface [4,5] (Figure 8).

Taken together, SEM results corroborate the gravimetric and spectroscopic findings: all three strains produced detectable morphological alterations in both polymers without abiotic pretreatment. Degradation proceeds predominantly via surface biofilm activity, with incorporation of hydroxyl and ether functional groups hypothesized to result from alkane-hydroxylase-mediated oxidation (AlkB system), without detectable deep carbonyl accumulation under the in vitro conditions of this study.

### 3.9. Scientific Novelty and Microbial Context

To our knowledge, there are few reports of PE-degrading capacity without pretreatment for isolates of *E. hormaechei* and *A. pittii* originating from the *G. mellonella* gut microbiome, which expands current knowledge on the functional diversity of this microbial ecosystem. The identification of these genera underscores the relevance of Enterobacter and, especially, Acinetobacter in the transformation of polymers and complex hydrophobic compounds. It is important to clarify that, although recent studies have documented the prevalence of Acinetobacter as a versatile colonizer in the food industry, where its enzymatic capacity (lipases and proteases) contributes to raw milk spoilage [35], its occurrence in this niche suggests a functional divergence toward the catabolism of synthetic polymers. This metabolic plasticity indicates that the microbiota of plastic-specialized insects constitutes a promising source of bacterial consortia capable of adapting their enzymatic machinery to overcome the high activation energy of carbon–carbon σ bonds. In this work, results for each bacterium are presented separately, but it is worth recalling that these organisms appear to act synergistically with the insect, which could substantially enhance their potential, as discussed previously.

#### 3.9.1. Methodological Validation: DCPIP Assay and Mass Loss

Validation of the degradative process relied on the colorimetric 2,6-dichlorophenolindophenol (DCPIP) assay, a high-throughput screening tool whose effectiveness lies in its capacity to act as an electron acceptor. The chromatic transition of the indicator (from blue to colorless) reflects the activity of the respiratory chain and bacterial dehydrogenases as they metabolize PE degradation intermediates. As discussed in Section 3.5, DCPIP consumption was high and similar among the three strains, while gravimetric mass loss showed greater discrimination among them, with an inverse pattern between the two variables; given the small number of strains (n = 3), no formal correlation was established between the two assays. This dissociation is consistent with the nature of each method: DCPIP reflects reduction kinetics attributable to general dehydrogenase/redox activity toward the polymer as a substrate, while gravimetry captures net cumulative mass loss over the long term, an outcome additionally influenced by cell adherence and biofilm formation. Both assays should therefore be interpreted as complementary lines of evidence, not as quantitative cross-validation of one another; the protein-based nature of this enzymatic activity is explored directly by SDS-PAGE in Section 3.7.

#### 3.9.2. Proposed Metabolic Mechanism: The AlkB System and FTIR-ATR Analysis

FTIR-ATR analysis revealed the appearance of hydroxyl (–OH) and ether (C–O–C) groups in the absence of a detectable carbonyl band (Table 6) (Figure 6, Figure 7 and Figure 8) (Section 3.6), a pattern consistent with the hypothesis that the alkane catabolic pathway, if mediated by the alkane hydroxylase (AlkB) system, an integral membrane oxygenase with a non-heme iron center, would initiate terminal oxidation of the inert hydrocarbon chain. Under this proposed mechanism, the AlkB enzyme would introduce molecular oxygen to generate primary alcohols, which would subsequently be transformed into fatty acids for entry into the β-oxidation pathway, without detectable accumulation of the carbonyl intermediate under the conditions of this assay. This radical oxygenation capacity has been reported indirectly in versatile environmental bacteria that, although frequently studied for their role in the hydrolysis of organic components in effluents [36] or dairy matrices [35], demonstrate an effective specialization for the chemical functionalization of hydrophobic backbones. It should be emphasized that this proposed AlkB-mediated mechanism is inferred from indirect spectroscopic and biochemical evidence; direct molecular or biochemical confirmation (e.g., genomic analysis, targeted PCR, proteomic analysis, or direct enzymatic assays), which were outside the objectives of the present study, remains an important direction for future work.

#### 3.9.3. Morphological Evidence and the Role of Biofilm (SEM and EPS)

Scanning electron microscopy (SEM) micrographs revealed severe physical degradation, manifested through surface pitting, longitudinal fractures, and localized erosion. These morphological alterations are the direct result of mature biofilm formation. The extracellular polymeric substance (EPS) matrix acts as a critical chemical interface that not only concentrates extracellular enzymes near the polymer surface but also accelerates natural changes in the material’s density and hydrophilicity [37]. By modifying surface roughness and reducing the original hydrophobic nature, EPS facilitates sustained mechanical and chemical fragmentation. Thus, the biofilm creates a microenvironment where radical-mediated oxidation occurs at a rate higher than abiotic degradation, enabling colonial anchoring and penetration into the polymer matrix.

### 3.10. Implications for Circular Biotechnology

The use of isolates from *G. mellonella* represents a significant advance toward the biotechnological valorization of plastic waste. Unlike techniques such as pyrolysis coupled with GC/MS, which are used strictly for monitoring and destructive analytical characterization of microplastics [38], these biological isolates enable biotransformation of the substrate into biomass or chemical precursors. The bacterial enzymatic system offers a sustainable alternative to the aggressive chemical extractions and acid digestions used to separate microplastics from industrial effluents [36], reducing the use of corrosive reagents. The ability of these strains to naturally increase the hydrophilicity of PE surpasses the slow environmental aging processes described in marine sediments [37], proposing a model for accelerated, controlled treatment under circular-economy principles.

## 4. Conclusions

The present study demonstrates that the dietary PE-induction protocol in *Galleria mellonella* larvae constitutes an effective in vivo enrichment strategy for recovering bacteria with polyethylene-degrading activity. The insect gut operates as a natural selection bioreactor that functionally concentrates the bacterial taxa with the greatest catabolic capacity toward PE. Through 16S rRNA gene sequencing, five isolates belonging to three species were identified: *Pseudomonas aeruginosa* (two isolates), *Enterobacter hormaechei* (one isolate), and *Acinetobacter pittii* (two isolates).

In the literature reviewed, there are few prior reports of PE-degrading activity without pretreatment documented individually for *E. hormaechei* and *A. pittii*. Activity was convergently assessed by four complementary methods: DCPIP-based redox/metabolic activity toward PE (>93% DCPIP reduction at day 7), a consistent, biologically coherent trend of gravimetric mass loss across strains that did not reach formal statistical significance at the available biological n (*E. hormaechei t*-test = 4.623, *p* = 0.136; *A. pittii t*-test = 2.845, *p* = 0.215; *P. aeruginosa t*-test = 1.652, *p* = 0.347), FTIR-ATR (changes in UI, HI, and COI), and SEM (active biofilm and surface erosion). The metabolic resistance test showed moderate thermoresistance and enzymatic inducibility under UV stress in *E. hormaechei* and *A. pittii*.

The finding of greatest biotechnological relevance is the demonstrated activity toward native PE without abiotic pretreatment, indicating that *E. hormaechei* and *A. pittii* possess the enzymatic machinery necessary to initiate the polymeric attack under real environmental conditions. The FTIR-ATR results—absence of deep carbonylic oxidation with the presence of hydroxyl and C–O-containing functionalities—are consistent with the hypothesis of AlkB system activation and β-oxidation of intermediates, a mechanism complementary to, and distinct from, the carbonylic oxidation attributed to host enzymes [8]; direct molecular or biochemical confirmation of this proposed pathway remains a direction for future work. The SDS-PAGE analyses provided direct evidence of the protein-based nature of the degradative mechanism.

## Figures and Tables

**Table 1:** Composition of the experimental diets administered to *G. mellonella* larvae. Treatment 3 used beeswax instead of honey as the lipid source.

Group	Diet	Addition	Total Feed (kg)
Control	300 g wheat bran, 160 g calf milk, 120 g wheat flour, 250 mL honey	—	0.83
Treatment 1	300 g wheat bran, 160 g calf milk, 120 g wheat flour, 250 mL honey	30 g LDPE powder (500 mesh)	0.86
Treatment 2	300 g wheat bran, 160 g calf milk, 120 g wheat flour, 250 mL honey	70 g LDPE powder (500 mesh)	0.90
Treatment 3	300 g wheat bran, 160 g calf milk, 120 g wheat flour, 250 mL beeswax	—	0.83

**Table 2:** Composition of the carbon-free liquid basal medium (CFLBM) used in the biodegradation assays.

Component	Concentration (g/L)
Na_2_HPO_4_	2.44
KH_2_PO_4_	1.56
(NH_4_)_2_SO_4_	2.00
NaCl	0.50
MgSO_4_·7H_2_O	0.20
FeCl_3_·6H_2_O	0.005
Trace element solution	1 mL/L

**Table 3:** Molecular identification of the five bacterial isolates by 16S rRNA gene sequencing and BLASTn analysis (GenBank, NCBI).

Isolate	Identified Species	BLAST Reference Accession	% Identity	Aligned Positions	E-Value	GenBank Accession
UNKNOWN0	*Pseudomonas aeruginosa*	CP012001.1	99.79	1451/1454	0.0	PZ794118
UNKNOWN8	*Pseudomonas aeruginosa*	CP012001.1	99.93	1470/1471	0.0	PZ794120
UNKNOWN1	*Enterobacter hormaechei*	CP017179.1	99.72	1454/1458	0.0	PZ794119
UNKNOWN9	*Acinetobacter pittii*	NR_117621.1	99.86	1460/1462	0.0	PZ794121
UNKNOWN10	*Acinetobacter pittii*	NR_117621.1	99.86	1454/1456	0.0	PZ794122

**Table 4:** DCPIP-based metabolic (redox) activity toward PE, expressed as percentage of DCPIP reduction.

Day	*P. aeruginosa* (%)	*E. hormaechei* (%)	*A. pittii* (%)
1	0.00	0.00	0.00
4	90.23	89.77	64.67
5	92.27	93.00	79.03
6	92.50	95.17	91.37
7	96.07	95.93	93.80

**Table 5:** Gravimetric consumption percentage and daily degradation rate (mg/day) for PE and OXO films after 45 days of incubation.

Bacterial Strain	% PE Consumption	% OXO Consumption	PE Rate (mg/day)	OXO Rate (mg/day)
Control	0.00	0.00	0.000	0.000
*P. aeruginosa*	1.29	0.25	0.287	0.073
*E. hormaechei*	2.52	1.29	0.560	0.287
*A. pittii*	3.13	1.37	0.696	0.304

**Table 6:** Spectroscopic degradation indices (UI, HI, COI) for control and bacteria-treated PE and OXO samples.

Sample	Score	VI	HI	COI	Similarity
Control PE	871	0.15	0.73	0.29	High
*P. aeruginosa* (PE)	862–865	0.35	—	—	High
*E. hormaechei* (1PE)	836–848	0.09	0.71	0.17	Moderate
*E. hormaechei* (2PE)	836–848	0.15	—	0.92	Moderate
*A. pittii* (1PE)	836–848	0.13	0.45	0.19	Moderate
*A. pittii* (2PE)	836–848	0.12	0.54	0.11	Moderate
Control OXO	859	0.11	—	—	High
*P. aeruginosa* (1OXO)	863–866	0.26	—	—	High
*E. hormaechei* (1OXO)	850–862	0.18	0.71	0.05	Mod–High
*E. hormaechei* (2OXO)	850–862	0.26	1.25	0.10	Mod–High
*A. pittii* (1OXO)	850–862	0.35	0.81	0.10	Mod–High
*A. pittii* (2OXO)	850–862	0.26	0.68	0.08	Mod–High

## Data Availability

The 16S rRNA gene sequences generated and analyzed during this study have been deposited in the NCBI GenBank database under accession numbers PZ794118 through PZ794122. All other supporting experimental data (including DCPIP kinetics, gravimetric weights, and FTIR-ATR raw spectra) are available within the article, its accompanying Supplementary Materials, or can be obtained upon reasonable request from the corresponding authors.

## Supplementary Materials

The following supporting information can be downloaded at: https://www.mdpi.com/article/10.3390/polym18182309/s1, Table S1: Raw DCPIP absorbance values (600 nm) and relative PE consumption percentage (%) at each incubation time point for three bacterial strains and controls; Table S2: ln(At/A0) values for first-order kinetic analysis of DCPIP reduction. A0 = 3.000 (initial absorbance, day 1); Table S3: First-order kinetic parameters obtained by linear regression of ln(At/A0) vs. incubation day; Table S4: Relative DCPIP consumption (%) under normal, boiling (100 °C/15 min), and UV irradiation (15 min) treatments for three bacterial strains; Table S4-raw: Complete raw dataset for the DCPIP metabolic resistance test: treatment × biological replicate × day × absorbance (600 nm), for each bacterial strain and the sterile control, underlying the averaged values reported in Table S4; Table S4B: Consolidated two-way repeated-measures ANOVA (n = 2 replicates) for relative PE consumption on experimentally measured days (Days 1, 3, and 5); Table S5: Individual replicate initial and final weights (g) of PE films and calculated mass loss (%) after 45 days of incubation; Table S5B: Individual replicate initial and final weights (g) of oxo-degradable (OXO) films and calculated mass loss (%) after 45 days of incubation. New in this revision—reconstructed from the raw weight records in “Absorbancias_con_T_Student.xlsx” (sheet 5), previously not tabulated in the Supplementary Material; Table S6: Summary statistics and Student’s t-test results for gravimetric PE degradation (% mass loss) after 45 days, compared to abiotic control; Table S7: Summary statistics and Student’s t-test results for gravimetric OXO biodegradable film degradation (% mass loss) after 45 days. Corrected in this revision—see note below; Table S8: Comparison of DCPIP-based metabolic activity (% consumption, day 7) and gravimetric PE mass loss (mean ± SD, %) for the three bacterial species; Table S9: Kruskal–Wallis test results for DCPIP relative consumption among groups [15,26].

## Abbreviations

The following abbreviations are used in this manuscript:

AlkB	Alkane hydroxylase
CFLBM	Carbon-free liquid basal medium
COI	Carbon-oxygen index
DCPIP	2,6-Dichlorophenolindophenol
EPS	Extracellular polymeric substance
FTIR-ATR	Attenuated total reflectance Fourier-transform infrared spectroscopy
HI	Hydroxyl index
LDPE	Low-density polyethylene
MCO	Multicopper oxidase
OXO	Oxo-biodegradable polyethylene
PE	Polyethylene
SDS-PAGE	Sodium dodecyl sulfate–polyacrylamide gel electrophoresis
SEM	Scanning electron microscopy
SENACYT	Secretaría Nacional de Ciencia, Tecnología e Innovación
TLA	Three-letter acronym
UI	Unsaturation index
UNACHI	Universidad Autónoma de Chiriquí
