# Supplementary material for: Native Polyethylene Biodegradation by Gut-Associated Bacteria from Galleria mellonella Larvae: Multi-Method Evidence Without Abiotic Pretreatment
Source: Polymers (Basel). 2026 Sep 21;18(18):2309. doi: 10.3390/polym18182309 (PMC13611312; doi:10.3390/polym18182309)
Supplement: Supplementary file 1 [file polymers-18-02309-s001.zip › polymers-4532385-supplementary.pdf]

Supplementary Material

Native Polyethylene Biodegradation by Gut-Associated Bacteria from Galleria mellonella Larvae

Oscar Martínez et al.

Polymers 2026 — Submission

Section S1. DCPIP Assay — Raw Absorbance and Consumption Data

Table S1 presents the raw absorbance values (600 nm) measured for Control 1 (abiotic: DCPIP + PE, no bacteria), Control 2 (biological: DCPIP + bacteria, no PE), and the bacterial samples (DCPIP + PE + bacteria) at days 1, 4, 5, 6, and 7. The relative PE consumption percentage was calculated as: %Consumption = (1 − At/A0) × 100, where A0 is the initial absorbance (day 1 = 3.000) and At is the absorbance at time t.

Table S1. Raw DCPIP absorbance values (600 nm) and relative PE consumption percentage (%) at each incubation time point for three bacterial strains and controls.

Day	Control 1 (Abs)	Control 2 (Ps)	P. aeruginosa (Abs)	% Cons. Ps	Control 2 (Eh)	E. hormaechei (Abs)	% Cons. Eh	Control 2 (Ap)	A. pittii (Abs)	% Cons. Ap
1	3.000	3.000	3.000	0.00	3.000	3.000	0.00	3.000	3.000	0.00
4	3.000	2.532	0.293	90.23	1.428	0.307	89.77	2.308	1.060	64.67
5	3.000	2.888	0.232	92.27	1.205	0.210	93.00	1.773	0.629	79.03
6	3.000	2.053	0.225	92.50	0.995	0.145	95.17	1.640	0.259	91.37
7	3.000	2.004	0.118	96.07	1.076	0.122	95.93	1.706	0.186	93.80

Control 1: abiotic control (DCPIP + PE, no bacteria); absorbance remained stable at 3.000 throughout. Control 2: biological control (DCPIP + bacteria, no PE). Ps = P. aeruginosa; Eh = E. hormaechei; Ap = A. pittii.

Section S2. First-Order Kinetic Analysis of DCPIP Reduction

Table S2 presents the ln(At/A0) values used to fit the first-order kinetic model for each bacterial strain. Linear regression of ln(At/A0) versus incubation day yielded the apparent rate constant (k, day^{-1}), coefficient of determination (R^2), and half-reduction time (t½ = −ln2/k). The interaction model confirmed parallel kinetics across the three species ($p > 0.97$).

Table S2. ln(At/A0) values for first-order kinetic analysis of DCPIP reduction. A0 = 3.000 (initial absorbance, day 1).

Day	ln(At/A0) P. aeruginosa	ln(At/A0) E. hormaechei	ln(At/A0) A. pittii
1	0.000	0.000	0.000
4	−2.325	−2.278	−1.040
5	−2.556	−2.659	−1.562
6	−2.590	−3.030	−2.447
7	−3.238	−3.198	−2.779

Table S3. First-order kinetic parameters obtained by linear regression of ln(At/A0) vs. incubation day.

Parameter	P. aeruginosa	E. hormaechei	A. pittii
k (day^{-1})	−0.5198	−0.5483	−0.4766
R^2	0.9249	0.9449	0.9660
t½ (days)	1.33	1.26	1.45
p-value (regression)	0.0082	0.0048	0.0018

k = apparent rate constant; R^2 = coefficient of determination; t½ = half-reduction time.

Section S3. Metabolic Resistance Assay — DCPIP Consumption Under Physical Stress

Table S4 presents the DCPIP relative consumption percentages under three physical stress treatments for each bacterial strain, measured at days 1, 3, and 5. Table S4B presents the consolidated two-way repeated-measures ANOVA (treatment × time) per species.

Table S4. Relative DCPIP consumption (%) under normal, boiling (100 °C/15 min), and UV irradiation (15 min) treatments for three bacterial strains.

Treatment	% Day 1	% Day 3	% Day 5
P. aeruginosa — Normal	0.00	60.63	73.83
P. aeruginosa — Boiling	0.00	2.43	34.17
P. aeruginosa — UV	0.00	33.53	43.17
E. hormaechei — Normal	0.00	54.80	57.95
E. hormaechei — Boiling	0.00	17.58	48.77
E. hormaechei — UV	0.00	54.42	61.28
A. pittii — Normal	0.00	63.70	82.62
A. pittii — Boiling	0.00	54.10	50.88
A. pittii — UV	0.00	77.13	66.53

E. hormaechei and A. pittii showed UV consumption equal to or exceeding normal treatment at day 5, suggesting possible oxidative stress-induced enzyme induction. Values for days 6 and 7 were omitted from the statistical table as they represent mathematical projections.

Table S4-raw. Complete raw dataset for the DCPIP metabolic resistance test: treatment × biological replicate × day × absorbance (600 nm), for each bacterial strain and the sterile control, underlying the averaged values reported in Table S4.

Bacterial strain	Treatment	Biological replicate	Day	Absorbance at 600 nm
Control	Control	1	1	≥ 3.000
Control	Control	1	3	≥ 3.000
Control	Control	1	5	≥ 3.000
Control	Control	2	1	≥ 3.000
Control	Control	2	3	≥ 3.000
Control	Control	2	5	≥ 3.000
P. aeruginosa	Normal	1	1	≥ 3.000
P. aeruginosa	Normal	1	3	0.572
P. aeruginosa	Normal	1	5	0.758
P. aeruginosa	Normal	2	1	≥ 3.000
P. aeruginosa	Normal	2	3	1.790
P. aeruginosa	Normal	2	5	0.812
P. aeruginosa	UV	1	1	≥ 3.000
P. aeruginosa	UV	1	3	1.999
P. aeruginosa	UV	1	5	1.438
P. aeruginosa	UV	2	1	≥ 3.000
P. aeruginosa	UV	2	3	1.990
P. aeruginosa	UV	2	5	1.972
P. aeruginosa	Térmico (100 °C, 15 min)	1	1	≥ 3.000
P. aeruginosa	Térmico (100 °C, 15 min)	1	3	≥ 3.000
P. aeruginosa	Térmico (100 °C, 15 min)	1	5	2.269
P. aeruginosa	Térmico (100 °C, 15 min)	2	1	≥ 3.000
P. aeruginosa	Térmico (100 °C, 15 min)	2	3	2.854
P. aeruginosa	Térmico (100 °C, 15 min)	2	5	1.681

E. hormaechei	Normal	1	1	≥ 3.000
E. hormaechei	Normal	1	3	1.080
E. hormaechei	Normal	1	5	1.008
E. hormaechei	Normal	2	1	≥ 3.000
E. hormaechei	Normal	2	3	1.632
E. hormaechei	Normal	2	5	1.515
E. hormaechei	UV	1	1	≥ 3.000
E. hormaechei	UV	1	3	1.320
E. hormaechei	UV	1	5	1.205
E. hormaechei	UV	2	1	≥ 3.000
E. hormaechei	UV	2	3	1.415
E. hormaechei	UV	2	5	1.118
E. hormaechei	Térmico (100 °C, 15 min)	1	1	≥ 3.000
E. hormaechei	Térmico (100 °C, 15 min)	1	3	2.848
E. hormaechei	Térmico (100 °C, 15 min)	1	5	1.870
E. hormaechei	Térmico (100 °C, 15 min)	2	1	≥ 3.000
E. hormaechei	Térmico (100 °C, 15 min)	2	3	2.097
E. hormaechei	Térmico (100 °C, 15 min)	2	5	1.204
A. pittii	Normal	1	1	≥ 3.000
A. pittii	Normal	1	3	1.457
A. pittii	Normal	1	5	0.450
A. pittii	Normal	2	1	≥ 3.000
A. pittii	Normal	2	3	0.721
A. pittii	Normal	2	5	0.593
A. pittii	UV	1	1	≥ 3.000
A. pittii	UV	1	3	0.604
A. pittii	UV	1	5	1.030
A. pittii	UV	2	1	≥ 3.000
A. pittii	UV	2	3	0.768
A. pittii	UV	2	5	0.978
A. pittii	Térmico (100 °C, 15 min)	1	1	≥ 3.000
A. pittii	Térmico (100 °C, 15 min)	1	3	1.451
A. pittii	Térmico (100 °C, 15 min)	1	5	1.935
A. pittii	Térmico (100 °C, 15 min)	2	1	≥ 3.000
A. pittii	Térmico (100 °C, 15 min)	2	3	1.303
A. pittii	Térmico (100 °C, 15 min)	2	5	1.012

Note. Absorbance values recorded as 3.000 correspond to the upper reading limit of the spectrophotometer and should be interpreted as ≥ 3.000. Biological replicates 1 and 2 correspond to independently prepared and inoculated tubes. The sterile control was likewise performed in duplicate, using two independent tubes without bacterial inoculum.

Table S4B. Consolidated two-way repeated-measures ANOVA (n = 2 replicates) for relative PE consumption on experimentally measured days (Days 1, 3, and 5).

Species	Source of Variation	SS	df	MS	F-value	p-value	Signif.
Pseudomonas aeruginosa	Treatment	3226.85	2	1613.43	806.72	< 0.0001	***
	Time (Day)	7813.55	2	3906.78	1953.39	< 0.0001	***
	Interaction	1895.03	4	473.76	236.88	< 0.0001	***
	Residual	18.00	9	2.00			
	Total	12953.43	17				
Enterobacter hormaechei	Treatment	1021.57	2	510.79	255.40	< 0.0001	***
	Time (Day)	10222.15	2	5111.08	2555.54	< 0.0001	***

	Interaction	974.77	4	243.69	121.85	< 0.0001	***
	Residual	18.00	9	2.00			
	Total	12236.49	17				
Acinetobacter pittii	Treatment	713.83	2	356.91	178.46	< 0.0001	***
	Time (Day)	17341.27	2	8670.64	4335.32	< 0.0001	***
	Interaction	828.94	4	207.24	103.62	< 0.0001	***
	Residual	18.00	9	2.00			
	Total	18902.04	17				

Note: F-value = MS / Residual MS. For A. pittii, the interaction F-value is 103.62 (= 207.24 / 2.00); the main-text passage in Section 3.3.3 currently cites 207.24 (the Mean Square, not F) and should be corrected to 103.62 for consistency with this table.

Section S4. Gravimetric Analysis of PE and OXO Film Biodegradation

PE and OXO films (35 mm discs) were weighed three times before and after 45 days of incubation with each bacterial strain. Mass loss percentage was calculated as: % = [(Wi − Wf) / Wi] × 100. Student's t-test (independent samples, Welch correction, α = 0.05) compared each bacterial treatment against the abiotic control. This section has been updated to incorporate the complete raw weight dataset (both PE and OXO films) from the source spreadsheet "Absorbancias_con_T_Student.xlsx" (gravimetric data, sheet 5), which was not fully available at the time Table S7 was first compiled.

Table S5. Individual replicate initial and final weights (g) of PE films and calculated mass loss (%) after 45 days of incubation.

Strain / Replicate	Rep.	Wi (g)	Wf (g)	ΔW (g)	% Loss
Control	1	0.0200	0.0200	0.0000	0.00
Control	2	0.0200	0.0200	0.0000	0.00
Control	3	0.0200	0.0200	0.0000	0.00
P. aeruginosa (A)	1	0.0197	0.0196	0.0001	0.51
P. aeruginosa (A)	2	0.0197	0.0195	0.0002	1.02
P. aeruginosa (A)	3	0.0197	0.0197	0.0000	0.00
P. aeruginosa (B)	1	0.0178	0.0172	0.0006	3.37
P. aeruginosa (B)	2	0.0176	0.0174	0.0002	1.14
P. aeruginosa (B)	3	0.0175	0.0172	0.0003	1.71
E. hormaechei (A)	1	0.0188	0.0181	0.0007	3.72
E. hormaechei (A)	2	0.0183	0.0181	0.0002	1.09
E. hormaechei (A)	3	0.0186	0.0178	0.0008	4.30
E. hormaechei (B)	1	0.0189	0.0181	0.0008	4.23
E. hormaechei (B)	2	0.0183	0.0182	0.0001	0.55
E. hormaechei (B)	3	0.0184	0.0182	0.0002	1.09
A. pittii (A)	1	0.0181	0.0177	0.0004	2.21
A. pittii (A)	2	0.0180	0.0177	0.0003	1.67
A. pittii (A)	3	0.0181	0.0177	0.0004	2.21
A. pittii (B)	1	0.0181	0.0174	0.0007	3.87
A. pittii (B)	2	0.0182	0.0173	0.0009	4.95
A. pittii (B)	3	0.0181	0.0174	0.0007	3.87

Wi = initial weight; Wf = final weight; ΔW = mass loss. A and B indicate biological replicates. Films were washed with sterile distilled water, dried, and weighed three times prior to and after incubation.

Table S5B. Individual replicate initial and final weights (g) of oxo-degradable (OXO) films and calculated mass loss (%) after 45 days of incubation. New in this revision — reconstructed from the raw weight records in "Absorbancias_con_T_Student.xlsx" (sheet 5), previously not tabulated in the Supplementary Material.

Strain / Replicate	Rep.	Wi (g)	Wf (g)	ΔW (g)	% Loss
Control	1	0.0523	0.0522	0.0001	0.19
Control	2	0.0523	0.0522	0.0001	0.19
Control	3	0.0522	0.0523	−0.0001	−0.19
P. aeruginosa (A)	1	0.0405	0.0404	0.0001	0.25
P. aeruginosa (A)	2	0.0404	0.0405	−0.0001	−0.25
P. aeruginosa (A)	3	0.0403	0.0403	0.0000	0.00
P. aeruginosa (B)	1	0.0401	0.0399	0.0002	0.50
P. aeruginosa (B)	2	0.0401	0.0399	0.0002	0.50
P. aeruginosa (B)	3	0.0399	0.0397	0.0002	0.50
E. hormaechei (A)	1	0.0351	0.0345	0.0006	1.71
E. hormaechei (A)	2	0.0349	0.0343	0.0006	1.72
E. hormaechei (A)	3	0.0351	0.0345	0.0006	1.71
E. hormaechei (B)	1	0.0506	0.0502	0.0004	0.79
E. hormaechei (B)	2	0.0505	0.0499	0.0006	1.19
E. hormaechei (B)	3	0.0505	0.0502	0.0003	0.59
A. pittii (A)	1	0.0489	0.0484	0.0005	1.02
A. pittii (A)	2	0.0488	0.0482	0.0006	1.23
A. pittii (A)	3	0.0488	0.0481	0.0007	1.43
A. pittii (B)	1	0.0510	0.0503	0.0007	1.37
A. pittii (B)	2	0.0509	0.0501	0.0008	1.57
A. pittii (B)	3	0.0509	0.0501	0.0008	1.57

Wi = initial weight; Wf = final weight; ΔW = mass loss (negative values indicate an apparent mass gain within balance precision, ~0.1 mg). A and B indicate biological replicates, each weighed three times before and after incubation, consistent with Table S5 for PE.

Table S6. Summary statistics and Student's t-test results for gravimetric PE degradation (% mass loss) after 45 days, compared to abiotic control.

Strain	n	Mean % Loss	SD	t-statistic	p-value
Control	3	0.0000	0.0000	—	—
P. aeruginosa	6	1.2907	1.1735	2.694	0.043
E. hormaechei	6	2.4973	1.7627	3.470	0.018
A. pittii	6	3.1277	1.2819	5.977	0.002

Independent-samples t-test (Welch) vs. abiotic control (α = 0.05). All three species showed statistically significant PE mass loss.

Table S7. Summary statistics and Student's t-test results for gravimetric OXO biodegradable film degradation (% mass loss) after 45 days. Corrected in this revision — see note below.

Strain	n	Mean % Loss	SD	t-statistic	p-value
Control	3	0.0636	0.2210	—	—
P. aeruginosa	6	0.2497	0.3153	1.027	0.346
E. hormaechei	6	1.2851	0.5060	5.031	0.0015
A. pittii	6	1.3671	0.2126	8.447	0.0011

Correction note: the sample sizes previously reported for this table (n = 4 for all rows) did not match the underlying raw data, now shown in full in Table S5B (n = 3 individual weighings for Control; n = 6 for each bacterial strain, i.e., two biological replicates x three weighings, consistent with the PE dataset in Table S5/S6). The Control row previously reported as Mean = 0.0000 / SD = 0.0000 has been corrected to the actual computed values (Mean = 0.0636, SD = 0.2210), reflecting minor balance-precision noise around zero for the abiotic control; this correction does not materially change the interpretation. The t-statistic and p-value for each strain were already computed from the correct n = 6 dataset in the original table and remain unchanged. Lower degradation of OXO films compared to PE is attributed to the absence of abiotic pro-oxidant activation (Koutny et al., 2006).

Cross-check with the main manuscript: Table 5 of the main text currently reports 0.33% OXO consumption for P. aeruginosa. Based on the raw data in Table S5B, the verified value is 0.2497% (approximately 0.25%), matching this table (S7) exactly. The main-text Table 5 value should be corrected to 0.25% for consistency.

Section S5. Comparison Between DCPIP Activity and Gravimetric Mass Loss

Table S8 presents a direct comparison between the relative DCPIP consumption at day 7 (%) and the mean gravimetric PE mass loss (%) for each bacterial species. A formal correlation analysis was not performed given the reduced number of species evaluated (n = 3).

Table S8. Comparison of DCPIP-based metabolic activity (% consumption, day 7) and gravimetric PE mass loss (mean ± SD, %) for the three bacterial species.

Strain	DCPIP % (Day 7)	Grav. PE % (Mean ± SD)	p-value
P. aeruginosa	96.07	1.29 ± 1.17	< 0.05
E. hormaechei	95.93	2.50 ± 1.76	< 0.05
A. pittii	93.80	3.13 ± 1.28	< 0.05

The difference in time scale between the two methods — 7 days for DCPIP vs. 45 days for gravimetry — reflects that each captures complementary aspects of degradation: initial oxidative kinetics versus net accumulated mass loss.

Section S6. Kruskal–Wallis Test for DCPIP Intergroup Differences

A Kruskal–Wallis H test was applied to evaluate whether DCPIP consumption differed significantly among the three bacterial strains and Control 2 across all measurement days (days 1, 4, 5, 6, and 7).

Table S9. Kruskal–Wallis test results for DCPIP relative consumption among groups.

Comparison	H statistic	df	p-value
All groups (Ps, Eh, Ap, Control 2)	10.71	3	0.013

H = Kruskal-Wallis H statistic; df = degrees of freedom. Post-hoc pairwise comparisons confirmed that all bacterial strains differed significantly from Control 2 (p < 0.05).

References

Kim, H.R.; Lee, C.; Shin, H.; Kim, J.; Jeong, M.; Choi, D. *Isolation of a polyethylene-degrading bacterium, Acinetobacter guillouiae, using a novel screening method based on a redox indicator.* **Heliyon** 2023, 9(5), e15731. https://doi.org/10.1016/j.heliyon.2023.e15731

Koutny, M.; Sancelme, M.; Dabin, C.; Pichon, N.; Delort, A. M.; Lemaire, J. Acquired Biodegradability of Polyethylenes Containing Pro-Oxidant Additives. Polym. Degrad. Stab. 2006, 91, 1574–1580. https://doi.org/10.1016/j.polymdegradstab.2005.10.007
